# Withaferin A Induces Cell Death Selectively in Androgen-Independent Prostate Cancer Cells but Not in Normal Fibroblast Cells

**DOI:** 10.1371/journal.pone.0134137

**Published:** 2015-07-31

**Authors:** Yukihiro Nishikawa, Daisuke Okuzaki, Kohshiro Fukushima, Satomi Mukai, Shouichi Ohno, Yuki Ozaki, Norikazu Yabuta, Hiroshi Nojima

**Affiliations:** 1 Department of Molecular Genetics, Research Institute for Microbial Diseases, Osaka University, 3–1 Yamadaoka, Suita City, Osaka, 565–0871, Japan; 2 DNA-chip Development Center for Infectious Diseases, Research Institute for Microbial Diseases, Osaka University, 3–1 Yamadaoka, Suita City, Osaka, 565–0871, Japan; Toho University School of Medicine, JAPAN

## Abstract

Withaferin A (WA), a major bioactive component of the Indian herb *Withania somnifera*, induces cell death (apoptosis/necrosis) in multiple types of tumor cells, but the molecular mechanism underlying this cytotoxicity remains elusive. We report here that 2 μM WA induced cell death selectively in androgen-insensitive PC-3 and DU-145 prostate adenocarcinoma cells, whereas its toxicity was less severe in androgen-sensitive LNCaP prostate adenocarcinoma cells and normal human fibroblasts (TIG-1 and KD). WA also killed PC-3 cells in spheroid-forming medium. DNA microarray analysis revealed that WA significantly increased mRNA levels of c-Fos and 11 heat-shock proteins (HSPs) in PC-3 and DU-145, but not in LNCaP and TIG-1. Western analysis revealed increased expression of c-Fos and reduced expression of the anti-apoptotic protein c-FLIP(L). Expression of HSPs such as HSPA6 and Hsp70 was conspicuously elevated; however, because siRNA-mediated depletion of HSF-1, an HSP-inducing transcription factor, reduced PC-3 cell viability, it is likely that these heat-shock genes were involved in protecting against cell death. Moreover, WA induced generation of reactive oxygen species (ROS) in PC-3 and DU-145, but not in normal fibroblasts. Immunocytochemistry and immuno-electron microscopy revealed that WA disrupted the vimentin cytoskeleton, possibly inducing the ROS generation, c-Fos expression and c-FLIP(L) suppression. These observations suggest that multiple events followed by disruption of the vimentin cytoskeleton play pivotal roles in WA-mediated cell death.

## Introduction

Withaferin A (WA), a major bioactive constituent of the Ayurvedic medicinal plant *Withania somnifera*, induces cell death in many tumor cells [1–3]. Specifically, WA dose-dependently induces apoptotic cell death mediated by the unfolded protein response (UPR), which is triggered by accumulation of misfolded proteins in the endoplasmic reticulum (ER), and it also causes apoptosis mediated by reactive oxygen species (ROS) in human renal cancer (Caki) cells [4], [5]. WA exerts potent antiproliferative effects by inhibiting Hsp90 chaperone activity, thereby inducing degradation of Hsp90 client proteins in pancreatic cancer cell lines Panc-1, MiaPaCa2, and BxPc3; this effect is reversed by the proteasome inhibitor MG132 [6]. WA suppresses expression of human papillomavirus E6/E7 oncogenes, restores p53 pathway activity, and induces apoptosis in cervical cancer CaSki cells [7]. WA causes cell-cycle arrest at G2/M phase in prostate cancer cell lines [8], and induces apoptosis in human melanoma cells by generating ROS and down-regulating Bcl-2 [9].

WA binds directly to vimentin by covalently modifying a cysteine residue (Cys328), causing vimentin filaments to aggregate and colocalize with F-actin and thereby disrupting the vimentin cytoskeleton [10], [11]. WA-induced vimentin aggregation is accompanied by changes in cell shape, decreased motility, and elevated vimentin phosphorylation at Ser38 [12]. These observations suggest that WA could be used to target metastatic tumor cells [12], [13]. Although WA inhibits the epithelial–mesenchymal transition (EMT) by suppressing vimentin function [14], the effect on vimentin alone cannot account for all of the WA-mediated subcellular events that lead to cell death. Indeed, WA also binds β-tubulin, inducing severe disruption of normal spindle morphology [15], and also disrupts the cellular organization of several other intermediate filament (IF) networks, including peripherin, neurofilament-triplet protein, and keratin [12]. Therefore, to fully understand the molecular mechanism of WA-induced cell death, we must identify additional molecular targets of WA.

The oncogenic transcription factor c-Fos regulates gene expression, in association with Jun or other basic leucine-zipper proteins, as a component of the activator protein 1 (AP-1) dimer complex. Although c-Fos exerts anti-apoptotic functions, recent reports suggest that it may also promote apoptosis [16], [17]. Activated c-Fos/AP-1 primes PC-3 and LNCaP prostate cancer (PCa) cells to undergo apoptosis by directly binding the promoter region of the anti-apoptotic gene c-FLIP(L), thereby repressing its transcription [18]. Apoptosis also requires activation of tumor necrosis factor (TNF)-related apoptosis-inducing ligand (TRAIL)/Apo-2L; this TRAIL-induced apoptosis is barely detectable in normal cells [18].

The pathogenesis of prostate tumors is initially androgen-dependent; however, many patients progress to metastatic castration-resistant (androgen-independent) PCa (mCRPC) [19]. Androgen-independent PCa cells (e.g., PC-3 and DU-145) exhibit stem-like characteristics, whereas androgen-responsive PCa cells (e.g., LNCaP) do not [20–22]. Annexin A1, a phospholipid-binding protein and regulator of glucocorticoid-induce [23]. NANOGP8, a retrogene encoding a NANOG1-like protein, also plays a role in regulating stem-like PCa cells [24]. Side-population cells from human PCa cell lines and xenograft tissues undergo more complete EMT and are more aggressive than homologous bulk-population cells [25]. Thus, the EMT appears to be closely associated with the development of mCRPC. To date, no drugs have been developed that effectively and potently kill mCRPCs but not normal cells.

In this study, we sought to investigate whether WA can kill mCRPCs but not normal cells, and if so, to identify the essential molecular target of WA. We found that WA (2 μM) selectively induced cell death in androgen-independent PC-3 and DU-145 PCa cells, but not in LNCaP androgen-responsive PCa cells or TIG-1 normal fibroblasts. DNA microarray and western analyses revealed novel molecular targets of WA that may define the distinct responses of these cell lines to WA. Because WA killed PC-3 cells in spheroid-forming cultures, we propose that WA may serve as a starting molecule for the development of drugs that induce cell death in cancer stem cells (CSCs).

## Results

### TIG-1 and LNCaP are less sensitive to WA than PC-3 and DU-145

**Fig 1:**
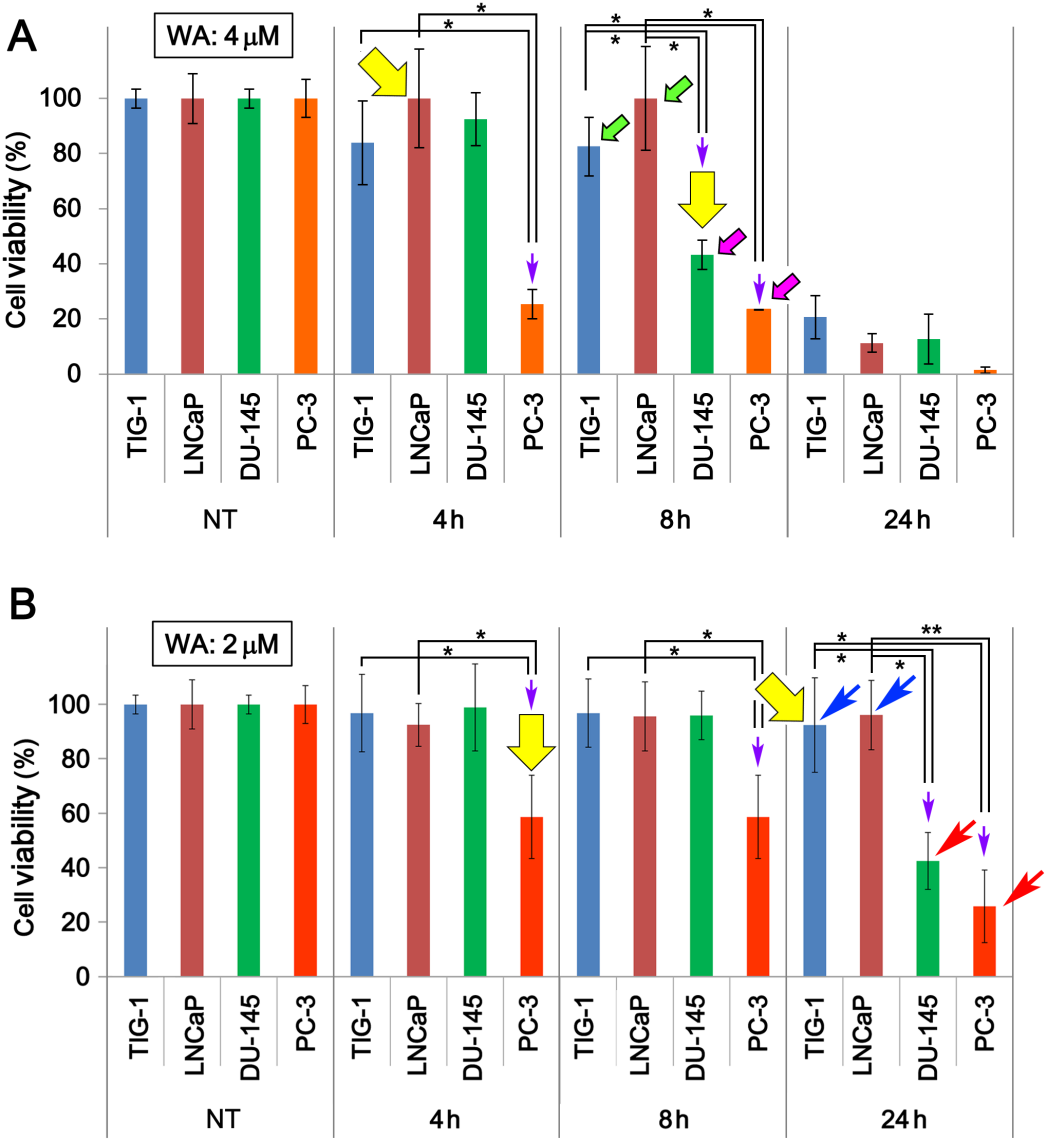
Cell viability of TIG-1, LNCaP, DU-145, and PC-3 cells after WA treatment. **(A, B)** Cell viability was measured at 4, 8, and 24 h after 2 μM (A) or 4 μM (B) WA treatment. NT, non-treated. Green and blue arrows indicate bars for surviving cells, whereas pink and red arrows indicate bars for cells that died under the same conditions. Yellow arrows indicate the samples used for DNA microarray analysis. Bars represent means ± SEM for three independent experiments. Purple arrows indicate significant reductions in cell viabilities (*, *P* < 0.05; **, *P* < 0.01).

We first found that WA effectively induced cell death in PC-3 and DU-145, but LNCaP were less sensitive to WA treatment (Fig 1). To determine whether normal human fibroblasts (TIG-1) also show altered resistance to WA, we compared the viability of TIG-1 exposed to 2 μM or 4 μM WA to the viabilities of LNCap, PC-3 and DU-145. Viability of PC-3 and DU-145 decreased significantly 8 h after 4 μM WA treatment (pink arrows in Fig 1A). By contrast, viability of TIG-1 remained high, at a level similar to that of LNCaP, up to 8 h after 4 μM WA treatment, when more than half of PC-3 and DU-145 had died (green arrows in Fig 1A). Cell viability of DU-145 was higher than that of PC-3 at 4, 8, and 24 h.

After treatment with 2 μM WA, viability of PC-3 was reduced at 4, 8, and 24 h, whereas DU-145 became inviable only at 24 h (red arrows in Fig 1B). By contrast, TIG-1 and LNCaP remained viable at 24 h (blue arrows in Fig 1B). Incubation of these cells for longer periods demonstrated that TIG-1 remained viable even at 96 h, whereas LNCaP became inviable at 72 h (S1A and S1C Fig), suggesting that 2 μM WA treatment does not induce the death of TIG-1 but the death of LNCaP was only delayed. Another human normal fibroblast line (KD) also showed resistance to 2 μM WA treatment (S1B Fig). Taken together, these results suggest that TIG-1 and LNCaP cells are more resistant to WA than PC-3 and DU-145 cells.

### Up-regulation of c-Fos after WA treatment correlates with cell viability

To identify genes that were up- or down-regulated in these cell lines following WA treatment, we examined the transcriptomes of TIG-1, LNCaP, PC-3, and DU-145 using Agilent SurePrint G3 Human Microarrays. We examined mRNA levels for PC-3 and TIG-1 at 4 h and 24 h, respectively, after 2 μM WA treatment, and for LNCaP and DU-145 at 4 h and 8 h, respectively, after 4 μM WA treatment (yellow arrows in Fig 1A and 1B). Fold changes in gene expression were determined by comparing hybridization signal intensities between samples treated with WA and those treated with dimethyl sulfoxide (solvent). S1 Table shows a list of differentially expressed genes, arranged in descending order of fold change in PC-3; all genes listed were also up-regulated by more than 4-fold in DU-145. Scatterplots indicate that the mRNA levels of analyzed genes were high enough (raw signal intensity > 10) to allow physiologically significant comparisons (S2 Fig).

**Fig 2:**
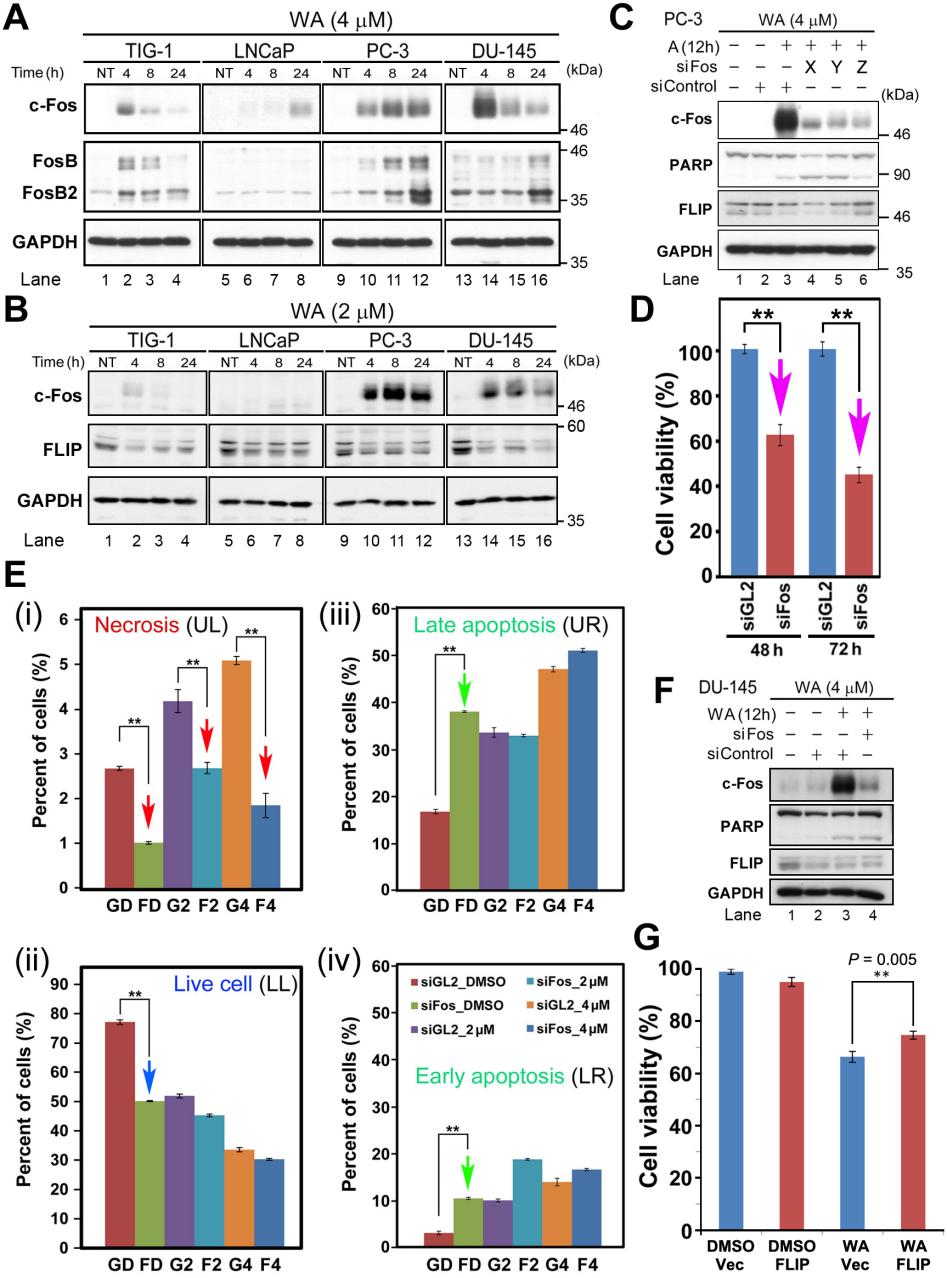
c-Fos and FLIP play a role in induction of cell death. (A, B) Western blot analysis to detect c-Fos (FosB) in TIG-1, LNCaP, DU-145, and PC-3 cells at 4, 8, and 24 h after treatment with 4 μM (A) or 2 μM (B) WA. NT, non-treated. (C) Western blot analysis to detect c-Fos, PARP, FLIP and GAPDH in PC-3 cells at 12 h after 4 μM WA treatment in the presence (+) or absence (-) of three different siRNAs (X–Z) from OriGene. (D) Viability of PC-3 cells after siFos treatment. Data are represented as means ± SEM of three independent experiments; pink arrows indicate a statistically significant reduction in cell number following siFos treatment (**, *P* < 0.01). (E) Population of apoptotic, necrotic, and live cells distinctly stained with Annexin V–EnzoGold, 7-AAD-Red, and GFP. Data are represented as means ± SEM of three independent experiments; red, blue, and green arrows indicate statistically significant changes (**, *P* < 0.01). (F) Western blot analysis of c-Fos, PARP, FLIP, and GAPDH in DU-145 at 12 h after 4 μM WA treatment in the presence (+) or absence (-) of siRNAs; siFos or siGL2 (siControl). (G) Viability of DU-145 cells after exogenous overexpression of pcDNA3-FLIP or vector alone in the presence of DMSO (solvent) or 4 μM WA.

Because overexpression of c-Fos induces apoptosis in human colorectal carcinoma cells [17], we focused on the c-Fos and FosB genes, which were conspicuously up-regulated in PC-3 and DU-145, but were weakly up-regulated in TIG-1 and even less so in LNCaP (S1 Table; pink and turquoise arrows in S2 Fig). In PC-3, these proteins were significantly up-regulated at 4 h after 4 μM WA treatment, followed by a gradual increase thereafter (Fig 2A). In DU-145, c-Fos was conspicuously up-regulated at 4 h, but its expression decreased gradually afterwards; a similar pattern was observed in TIG-1, although the up-regulation was less conspicuous than in DU-145 (Fig 2A). In LNCaP, the c-Fos level was very low, and was detectable only at 24 h. Notably, the ranked order of cell viability (see Fig 1A) was identical to the order of the c-Fos expression level at 8 h after 4 μM WA treatment (LNCaP > TIG-1 > DU-145 > PC-3). Moreover, after 2 μM WA treatment, a similar pattern of c-Fos up-regulation was observed in both PC-3 and DU-145; however, very little c-Fos expression was observed in TIG-1 and LNCaP (Fig 2B); thus, WA-induced c-Fos expression was correlated with cell viability. By contrast, FosB and FosB2 were not significantly up-regulated, and their expression levels were not correlated with cell viability (Fig 2A).

siRNA-mediated knockdown of c-Fos (siFos) rescues cell death in PC-3 [18]. Hence, we investigated what type of cell death (i.e., apoptosis or necrosis) is rescued by siFos. For these experiments, we used siFos_Z (Fig 2C). First, we confirmed in PC-3 that viability significantly decreased at 48 h and 72 h after WA treatment (Fig 2D). FACS analysis (S3 Fig) revealed that the rate of necrosis was lower in siFos-treated cells than in cells treated with siGL2 (negative control) 8 h after treatment with either DMSO alone or WA (2 μM or 4 μM), suggesting that overexpression of c-Fos induced by WA plays a role in inducing necrotic cell death (red arrows in Fig 2E-i). By contrast, under the same conditions, viability decreased (blue arrows in Fig 2E-ii) and the percentage of apoptotic cells increased (green arrows in Fig 2E-iii and 2E-iv). Thus, overexpression of c-Fos correlates with WA-induced cell death in PC-3. It remains elusive if c-Fos is involved in the determination of cell viability or just converts the cell death mode from apoptosis to necrosis without affecting the induction of cell death. We could not examine this in DU-145 because both siGL2 and siFos caused cell death at a similar level.

c-Fos exerts its proapoptotic function in PC-3 and LNCaP cells in part by repressing expression of c-FLIP(L), which we will refer to as ‘FLIP’ hereafter [18]. The FLIP level was reduced following WA treatment in all cells tested (Fig 2B); the reduction of FLIP was especially remarkable in DU-145, particularly at 24 h after 2 μM WA treatment (Fig 2B, lane 16), relative to the reductions in TIG-1, LNCaP, and PC-3 (Fig 2B, lanes 4, 8, and 12). This observation suggests that WA-mediated cell death occurs in part due to reduced expression of FLIP. Western blot analysis indicated that the level of FLIP was not reduced by siFos_Z treatment in PC-3 (Fig 2C, lane 6), whereas siFos_X or siFos_Y treatment slightly reduced the FLIP level (Fig 2C, lanes 4 and 5). In DU-145, the FLIP level was similarly reduced by treatment with siControl or siFos_Z (Fig 2F, lanes 3 and 4). These results suggest that modulation of the c-Fos level is somehow related to but does not directly affect the reduced FLIP level. Nonetheless, exogenous expression of FLIP in DU-145 rescued WA-induced cell death (Fig 2G). Taken together, these results suggest that WA caused two independent events, namely, an increase in the c-Fos level and a reduction in the FLIP level, to induce cell death.

### Expression of HSP genes is up-regulated after WA treatment

**Fig 3:**
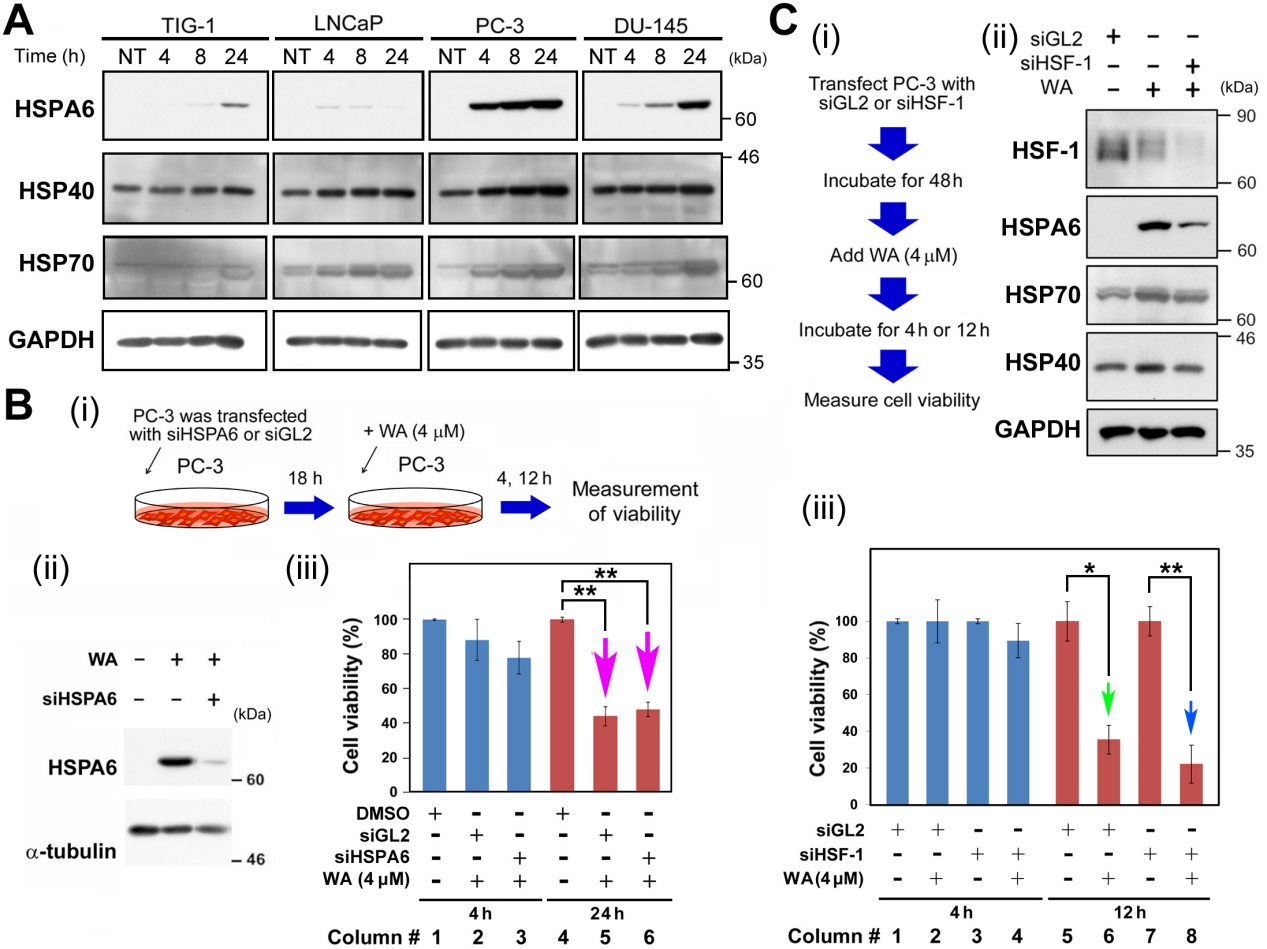
Expression of HSP genes is up-regulated after WA treatment. (A) Western blot analysis to detect HSPA6, HSP40, HSPA70, EGR-1, PAR-4, and GAPDH (loading control) in TIG-1, LNCaP, DU-145, and PC-3 cells at 0, 4, 8 and 24 h after 4 μM WA treatment. Arrow indicates the band for bona fide EGR-1. (B) Influence of siHSPA6 expression on PC-3 cell growth. (i) Schematic for this experiment. (ii) Western blot analysis to confirm the knockdown of HSPA6 protein by siHSPA6, relative to cells treated with siGL2 (negative control). (iii) Influence of siHSPA6 and siGL2 on PC-3 cell growth, with or without WA treatment. Arrow highlights the reduction in PC-3 cell growth. (C) Influence of siHSF-1 expression on PC-3 cell growth. (i) Schematics for this experiment. (ii) Western blot analysis to confirm the knockdown of HSP protein by siHSF-1, relative to cells treated with siGL2 (negative control), and to determine whether HSF-1 knockdown affected HSP protein levels by detection of HSPA6, HSP40, HSPA70, and GAPDH in PC-3 cells. (iii) Influence of siHSF-1 and siGL2 on PC-3 cell growth, with or without WA treatment. Arrow highlights the reduction in PC-3 cell growth. Data are represented as means ± SEM of three independent experiments; pink, green, and blue arrows indicate significant decreases in cell viabilities (*, *P* < 0.05; **, *P* < 0.01).

Next, we noticed that 11 heat-shock protein (HSP) genes (*HSPA6*, *DNAJA4*, *HMOX1*, *HSPA1B*, *HSPA1L*, *HSPA1A*, *DNAJB1*, *DNAJB4*, *HSPH1*, and *SERPINH1*) were among the 45 most up-regulated genes in PC-3 (S1 Table). HSPs are molecular chaperones that are subjected to rapid and strong induction by stress. In PC-3 and DU-145, HSPA6 was abruptly up-regulated 4 h after WA treatment, whereas in TIG-1 and LNCaP, the level of HSPA6 protein was very low (Fig 3A). By contrast, expression levels of DNAJA4 (HSP40 hereafter) and HSPA1B (HSP70 hereafter) exhibited similar gradual increases in these cells (Fig 3A).

To reduce the level of HSPA6 protein in WA-treated cells, we performed siRNA-mediated knockdown in PC-3 (Fig 3B-i). We confirmed successful knockdown by western blotting (Fig 3B-ii). However, we observed little change in cell viability in siHSPA6-treated cells relative to cells treated with a negative control RNA (siGL2) (red arrows in Fig 3B-iii), suggesting that overexpression of HSPA6 is not the cause of cell death after WA treatment.

We next performed siRNA-mediated knockdown of heat-shock factor protein 1 (HSF1), the major transcription factor involved in up-regulation of the HSF genes [26], using a similar experimental design (Fig 3C-i). Western analysis confirmed that HSF1 knockdown using siHSF1 decreased expression of both HSF1 and HSPA6 (Fig 3C-ii). By contrast, the levels of HSP40 and HSP70 were unaltered (Fig 3C-ii), probably due to the high baseline levels of endogenous HSP40 and HSP70 (see Fig 3A). Nonetheless, treatment of PC-3 cells with siHSF1 increased cell death (green and blue arrows in Fig 3C-iii), although the increase was not conspicuous. These results suggest that unknown proteins up-regulated by HSF1 protect PC-3 cells against cell death.

Four of the early growth response (EGR) genes were ranked among the top 40 WA-induced genes (S1 Table). The levels of EGR-1 and EGR-3 remained constant following WA treatment (S3 Fig); therefore, we ignored these proteins in subsequent analyses. Contrary to a previous report [27], prostate apoptosis response-4 (PAR-4) was not up-regulated after WA treatment at either the mRNA (green arrowheads in S1 Fig) or protein level (S3 Fig). Therefore, these genes may not be involved in determining cell viability (see Fig 1).

### WA induces the stress response initiated at the ER and mitochondria in prostate cancer cells

**Fig 4:**
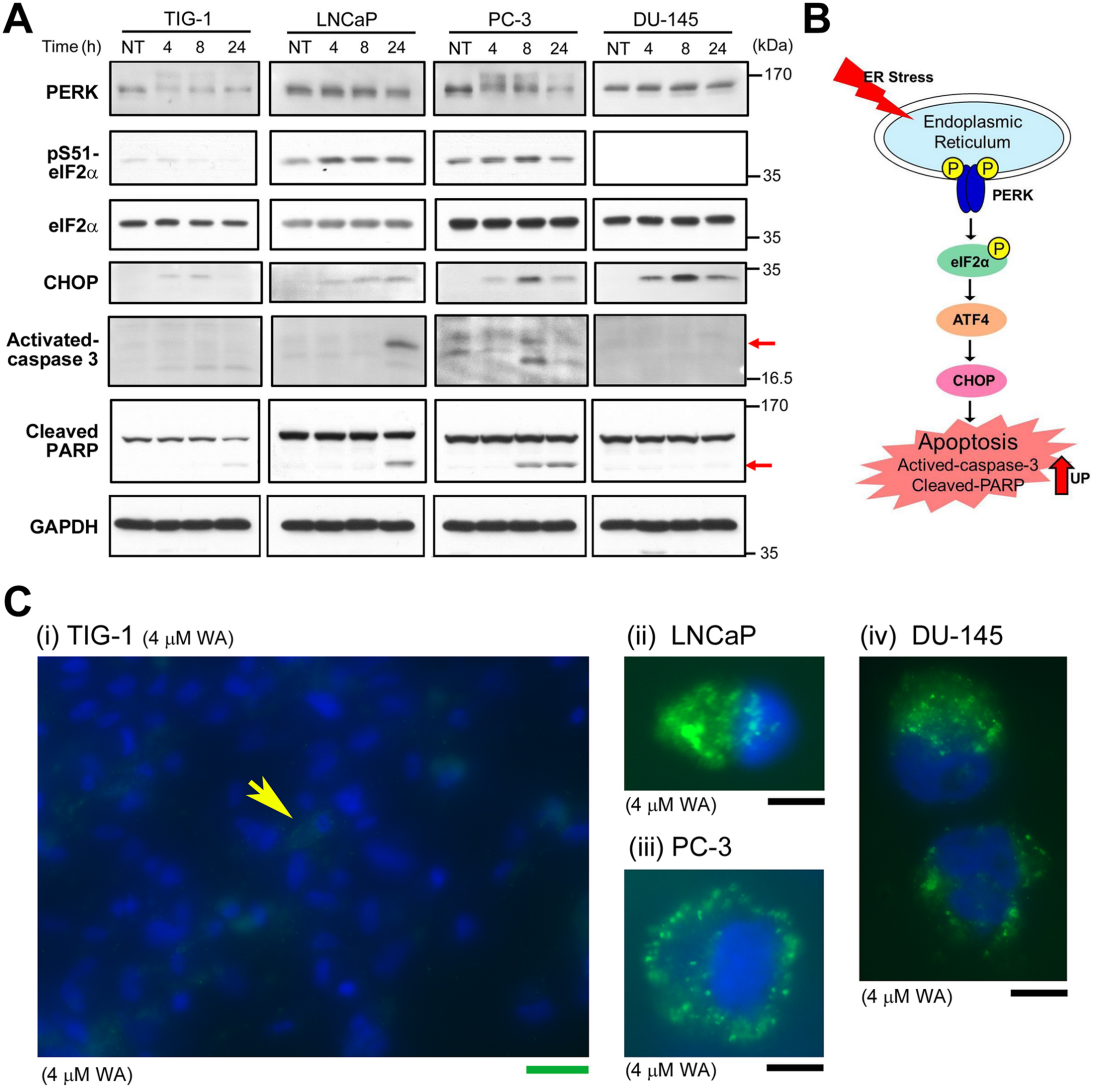
Expression profiles of ER stress–related proteins following WA treatment. (A) Western blot analysis to detect IRE-1α, PERK, pS51-eIF2α, CHOP, caspase 3, PARP, BiP, and GAPDH (loading control) in TIG-1, LNCaP, DU-145, and PC-3 cells at 4, 8, and 24 h after 4 μM WA treatment. NT: non-treated. (B) Schematic representation of the analyzed proteins in the apoptotic pathway induced by ER stress. (C) Typical fluorescence images of TIG-1 (i), LNCaP (ii), PC-3 (iii), and DU-145 (iv) that were treated with 4 μM WA for 24 h and subsequently treated with ROS detection reagents using the Image-iT LIVE Green ROS Detection Kit. Merged images are shown of cytoplasmic ROS signals (green) and nuclear DNA signals stained with Hoechst33342 (blue). Green bar, 100 μm. Black bar, 10 μm.

WA induces apoptosis mediated by ER stress in human renal carcinoma cells [4] and *Xenopus laevis* A6 kidney epithelial cells [28]. Gene Ontology analysis of the DNA microarray data (S5 Fig) using the NextBio system confirmed that the top-ranked enriched gene sets contained ER stress–related UPR genes (ID, GO:0006986; S4 Fig). To determine whether ER stress was induced to different degrees in TIG-1 and PC-3 after WA treatment, we performed western analysis at 2, 4, and 8 h after 4 μM WA treatment. In PC-3, induction of CHOP expression and activation of caspase 3 and PARP cleavage were observed, which suggests that the ER stress response via activation of CHOP is important (Fig 4). However, siRNA-mediated knockdown of CHOP suggest that CHOP does not play a role in the activation of caspase 3 (S6 Fig). By contrast, phosphorylation of eIF2α at Ser51 was inefficient, possibly due to weak activation of PERK; consequently, downstream events such as caspase 3 activation and PARP cleavage were barely detectable in TIG-1 (leftmost panels in Fig 4). This weak response to ER stress may explain why TIG-1 is resistant to WA treatment (see Fig 1B). In LNCaP, caspase 3 activation and PARP cleavage were not observed at 8 h; thus, in this cell line the ER stress response was activated at a level intermediate between those in TIG-1 and PC-3; the resistance of LNCaP to WA may be due to this delay in downstream events. However, although the level of CHOP was induced after WA treatment in DU-145, little caspase 3 activation and PARP cleavage was observed (rightmost panels in Fig 4). Thus, apoptotic cell death of DU-145 may be due primarily to increased c-Fos expression and reduced FLIP expression rather than the ER stress response (see Fig 3).

*Ashwagandha* leaf extract containing WA causes breast cancer cells to generate ROS, which is a stress response initiated at mitochondria [29]. We examined if treatment with WA also induces ROS generation in prostate cancer cells and normal fibroblasts by a fluorescence probe method. We first confirmed detection of ROS signals when cells were treated with *tert*-butyl hydroperoxide (TBHP), an inducer of ROS generation. All tested cells showed cytoplasmic signals (green) derived from ROS production (S7A Fig). Upon treatment with 4 μM WA for 24 h, only a low level of cytoplasmic ROS was detected in TIG-1 (Fig 4B-i) and KD (S7B Fig), which is similar to the finding of a previous report that TIG-3 generate little ROS after WA treatment [29]. By contrast, strong ROS signals were detected in LNCaP, PC-3, and DU-145 (Fig 4C-ii,-iii, and-iv). These results suggest that TIG-1 and KD, unlike LNCaP, PC-3, and DU-145, did not generate ROS after WA treatment, which may explain why TIG-1 and KD are resistant to WA.

### WA induces BAG3-mediated autophagy in PC-3 cells

**Fig 5:**
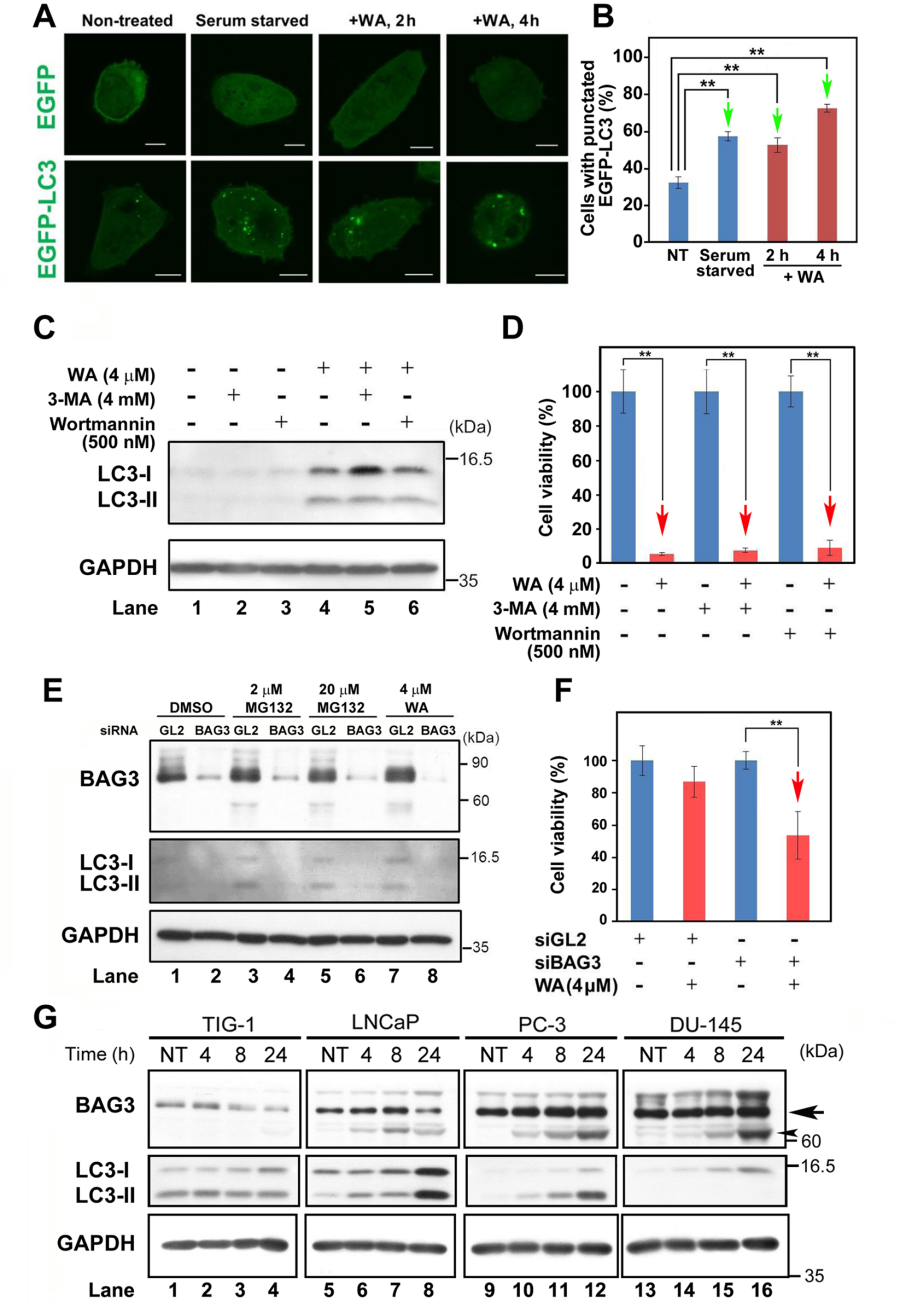
WA induces BAG3-mediated autophagy in PC-3 cells. A) Observation of EGFP and EGFP-LC3 signals by immunofluorescence microscopy. Bar, 10 μm. (B) Bar graphs showing the percentage of cells containing punctate EGFP-LC3; arrows show that the values gradually increased under the indicated conditions. Data are represented as the means ± SEM of three independent experiments; green arrows indicate statistically significant increases (**, *P* < 0.01). (C) Western blot analysis to detect LC-3 and GAPDH (loading control) in PC-3 under the indicated conditions. (D) Bar graphs showing cell viability (%) under the indicated conditions. (E) Western blot analysis to detect BAG3, LC-3, and GAPDH (loading control) in PC-3 cells in the presence of the indicated conditions of siBAG3 or siGL2 (negative control). (F) Bar graphs showing cell viability (%) at the indicated conditions. (D, F) Data are represented as means ± SEM of three independent experiments; red arrows indicate statistically significant reductions in cell viability (**, *P* < 0.01). (A–F) Samples were collected at 4 h after WA treatment. (G) Expression profiles of the autophagy-related proteins BAG3 and LC3 in PC-3 cells at 4, 8, and 24 h after treatment with 4 μM WA. NT: non-treated.

WA induces autophagy in breast cancer cells, but the detailed mechanism remains elusive [30]. Our DNA microarray data (S1 Table) indicated that the mRNA level of Bcl-2-associated athanogene 3 (BAG3), a member of the BAG co-chaperone family implicated in autophagy [31], is up-regulated after WA treatment. Thus, we investigated whether WA induced autophagy in PC-3 by expressing EGFP-tagged microtubule-associated protein light chain 3 (LC3), an autophagy marker that specifically labels autophagosomal membranes [32]. Indeed, EGFP-LC3 puncta appeared after 2 μM or 4 μM WA treatment (Fig 5A) at a frequency similar to that in serum-starved cells (Fig 5B).

To determine whether WA-mediated autophagy affects cell viability, we added pharmacological inhibitors of class III phosphatidylinositol 3-kinases, 3-methyladenine (3-MA) or wortmannin, which suppresses canonical autophagy, to PC-3 cells in the presence (+) or absence (−) of 4 μM WA treatment. Western analysis indicated that WA induced LC3 expression regardless of the presence of 3-MA or wortmannin (Fig 5C). Moreover, neither drug suppressed the reduction in cell viability (Fig 5D).

Because BAG3 activates noncanonical autophagy elicited by proteasome inhibitors such as MG132 [33], we investigated whether siRNA-mediated knockdown of BAG3 would affect PC-3 cell viability. siBAG3 successfully suppressed BAG3 expression compared to siGL2, a negative control (top panel of Fig 5E). As expected, LC3 expression was suppressed by siBAG3 after either treatment with DMSO, 2 μM MG132, 20 μM MG132, or 4 μM WA (middle panel of Fig 5E). Furthermore, cell viability was reduced by siBAG3 following WA treatment (Fig 5F), probably due to inhibition of the anti-apoptotic activity of BAG3. Taken together, these results suggest that WA-induced BAG3 mediates noncanonical autophagy, which protects PC-3 cells against cell death.

Western blot analysis showed that the intensity of the ~70 kDa band of BAG3 (arrow in Fig 5G) was decreased in TIG-1 at 24 h after WA treatment (Fig 5G, lane 4), whereas its level was already high (lanes 9 and 13) and unchanged after WA treatment in PC-3 (lanes 10–12) and DU-145 (lanes 14–16). Moreover, the ~62 kDa band of BAG3 (a putative processed form of BAG3) was conspicuously increased in PC-3 and DU-145 (arrowhead in Fig 5G); it remains elusive if 62 kDa BAG3 is more active than 70 kDa BAG3 or is inactive. These results indicate that the BAG3 protein level is higher in PC-3 and DU-145 than in TIG-1 and LNCaP. Nonetheless, the LC3 level was low in PC-3 and DU-145 (middle panels in Fig 5G), suggesting that a regulatory mechanism linking BAG3 activity and LC3-II production was altered in these cells. Indeed, a recent report showed that DU-145 cannot produce LC3-II due to a genetic impairment of the autophagy pathway [34]. By contrast, LC3 expression was high in TIG-1 and LNCaP (middle panels in Fig 5G). It remains to be analyzed if these results suggest that these cells are somehow protected from WA-induced cell death similar to the protection of Caco-2 cells from oxaliplatin-induced cell death [35].

### TIG-1 and PC-3 exhibit distinct patterns of subcellular vimentin localization following WA treatment

**Fig 6:**
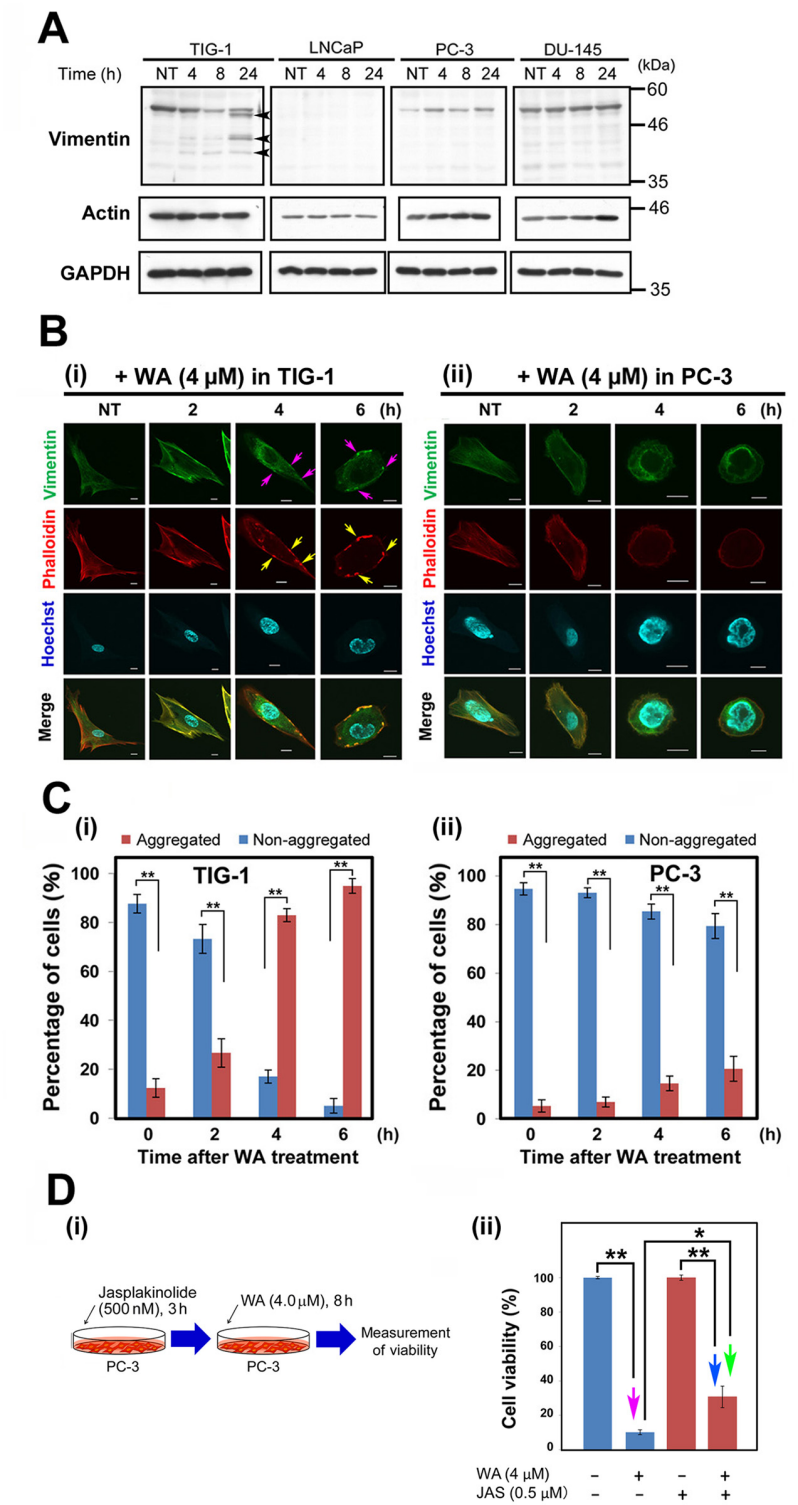
Protein levels and localizations of vimentin and F-actin following WA treatment. (A) Western blot analysis to detect vimentin and actin in TIG-1, LNCaP, PC-3, and DU-145 at 4, 8, and 24 h after treatment with 2 μM or 4 μM WA. NT: non-treated. (B) Typical images of TIG-1 (i) and PC-3 (ii) stained with anti-vimentin antibody, phalloidin (for F-actin), and Hoechst 33342 (for DNA) at 2, 4, and 6 (h) after WA treatment. NT: non-treated. Pink and yellow arrows indicated colocalized vimentin and F-actin aggregates. Merged images are shown in the bottom row. Bar, 10 μm. (C) Percentage of all observed cells containing dots of aggregated or non-aggregated vimentin are shown for TIG-1 (i) and PC-3 (ii). Cells harboring over 10 vimentin aggregates were scored as “aggregated”, whereas cells with non-aggregated vimentin were scored as “non-aggregated”. Data are represented as means ± SEM of three independent experiments; asterisks indicate statistically significant changes in cell viability (**, *P* < 0.01; ***, *P* < 0.001). (D) Jasplakinoloide (Jsp) did not influence PC-3 cell viability. (i) Schematic of the experimental design. (ii) Bar graphs showing cell viability (%) in the presence (+) or absence (-) of 4 μM WA or Jasp 8 h after the addition of WA. NT: non-treated. Bar graphs represent means ± SEM for three independent experiments. Pink, blue, and green arrows indicate statistically significant reductions in cell viability (*, *P* < 0.05; **, *P* < 0.01).

WA associates with vimentin and causes rapid reorganization of vimentin intermediate filaments (VIF) into perinuclear aggregates in BJ-5ta human fibroblasts [12]. Although mRNA levels of vimentin were high and unaltered after WA treatment in TIG-1, LNCaP, PC-3, and DU-145 (black arrows in S1 Fig), western analysis indicated that vimentin protein levels were elevated following WA treatment in PC-3 (Fig 6A). By contrast, vimentin level was already high before WA treatment, and remained high, in DU-145 (Fig 6A). In TIG-1, the vimentin level was reduced, with cleavage products of vimentin [10] evident at 24 h (arrowheads Fig 6A), whereas no vimentin band was detected in LNCaP (Fig 6A). These observations are consistent with the fact that PC-3, but not LNCaP, underwent the EMT to acquire vimentin expression.

Immunostaining at 2, 4, and 8 h after 4 μM WA treatment caused vimentin aggregation around the plasma membrane in TIG-1 (pink arrows, Fig 6B-i). By contrast, PC-3 exhibited a homogeneous distribution of vimentin, with no such aggregates (Fig 6B-i and 6B-ii); this suggests that vimentin expressed after the EMT in PC-3 serves a different function than it does in mesenchymal cells. Notably, the cytoplasmic volume of PC-3 was smaller than that of TIG-1. The percentage of cells harboring these aggregates increased conspicuously in TIG-1 (Fig 6C-i) relative to PC-3 (Fig 6C-ii).

Immuno-electron microscopy revealed vimentin signals at IFs in the cytoplasm in the absence of WA treatment (S8A Fig), confirming the mesenchymal origin of TIG-1. Notably, many vimentin signals shifted to the peripheral region of the cytoplasm near the plasma membrane, in a pattern that reflected the presence of the aforementioned vimentin aggregates (see Fig 6B-i), after 4 h of 4 μM WA treatment (S8B Fig). By contrast, only sparse vimentin signals were observed in PC-3 in the absence of WA treatment (S8C Fig); this observation is similar to those obtained by immunostaining (see Fig 6B-ii). Moreover, the vimentin signal was low in PC-3 treated with 4 μM WA for 4 h, at which point the cytoskeletal structure was disrupted (S8D Fig), suggesting that following an increase in the vimentin level after EMT, these proteins are not properly structured in PC-3 (S5 Fig) and that this disruption induces the expression of HSPs and c-Fos.

Next, we noticed that F-actin, another cytoskeletal component detected by phalloidin, was also aggregated in TIG-1 (yellow arrows in Fig 6B-i), and these bodies colocalized with the vimentin aggregates. By contrast, PC-3 cells (Fig 6B-ii) contained few F-actin aggregates. F-actin levels remained constant in these cells (Fig 6A). Moreover, when we added jasplakinolide, a cyclo-depsipeptide that promotes polymerization and stabilization of actin filaments [36], to PC-3 cells after 4 μM WA treatment for 8 h (Fig 6D-i), the reduction in cell viability (red arrow in Fig 6D-ii) was significantly suppressed (blue and green arrows in Fig 6D-ii), suggesting that WA induces abnormal F-actin polymerization and stabilization. Taken together, these results suggest that the distinct responses of the cytoskeletal architecture to WA treatment may explain, at least partly, the differences in viability between these cell lines (see Fig 1).

### PC-3 in serum-free medium exhibited similar chemoresistance to WA

**Fig 7:**
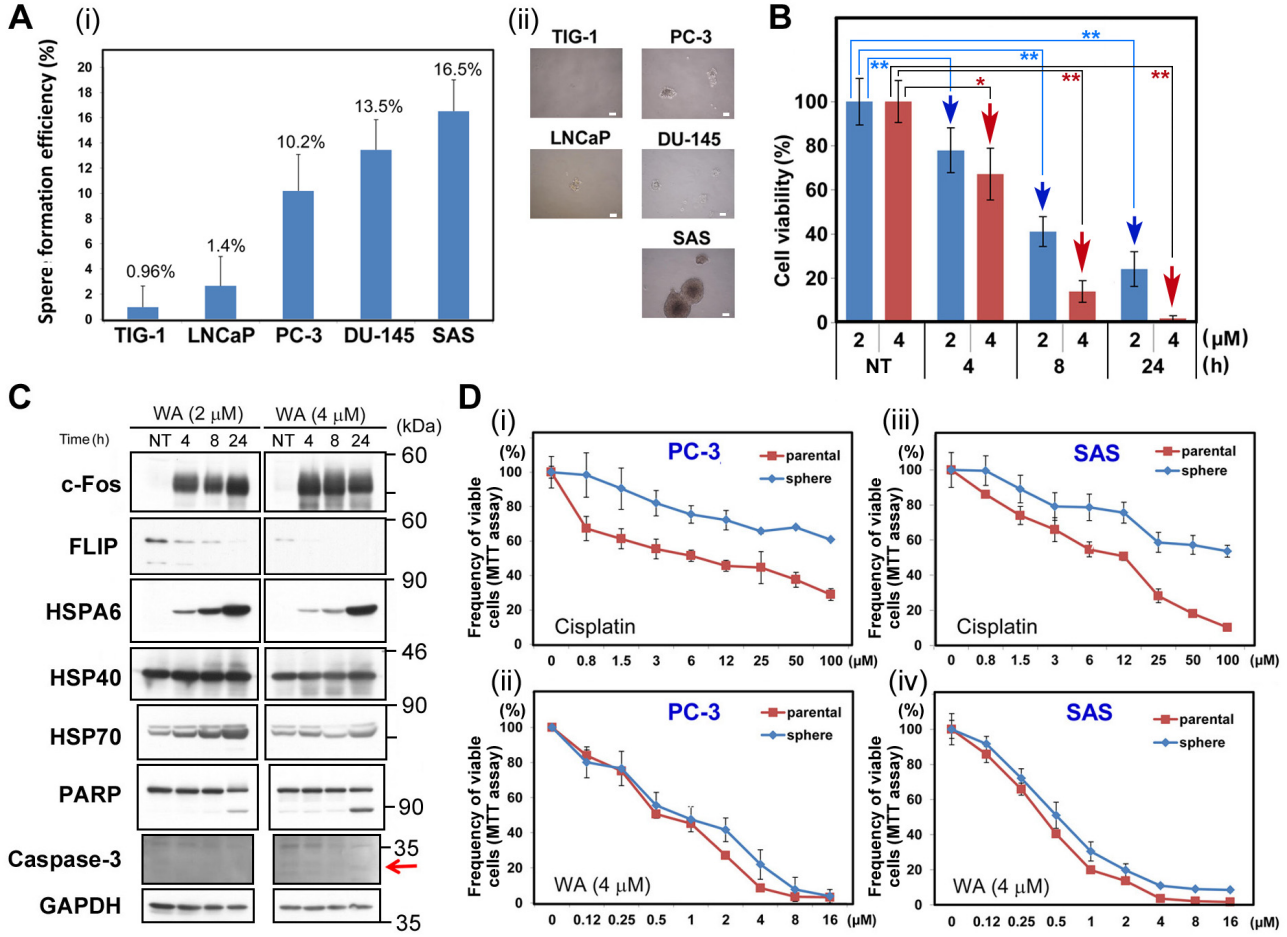
Sphere-forming PC3 and SAS cells had higher resistance to cisplatin, but not to WA, than adherent cells. (A) Frequency of spheres larger than 100 μm (i) and typical images (ii) after incubation of TIG-1, LNCaP, PC-3, DU-145, and SAS cells in sphere-formation medium for 10 days. (B) Viability of SAS cells after WA treatment. Bars represent means ± SEM of three independent experiments. Cell viability was measured at 4, 8, and 24 h after treatment with 2 μM (blue bars) or 4 μM (red bars) WA. NT, non-treated. Data are represented as means ± SEM of three independent experiments; red arrows indicate statistically significant reductions in cell viability (**, *P* < 0.01). (C) Western blot analysis to detect c-Fos, HSPA6, HSP40, HSP70, PARP, and GAPDH (loading control) in SAS cells at 4, 8, and 24 h after treatment with 2 μM or 4 μM WA. NT: non-treated. (D) Comparison of sensitivity to cisplatin between parental cells and spheres of PC-3 (i and ii) and SAS (iii and iv) after 48 h of incubation following treatment with indicated cisplatin concentrations.

The results shown above suggest that WA kills even the mCRPCs. Notably, PC-3 [21] and DU-145 [22] possess cancer stem cell (CSC)–like properties and form spheroids in serum-free medium (SFM) culture [37]. Indeed, PC-3 and DU-145 formed large (>100 μm) spheroids at rates similar to that of SAS (Fig 7A), a human tongue cancer cell line known to form large spheroids [37]. By contrast, TIG-1 and LNCaP failed to form large spheroids in SFM culture (Fig 7A). Furthermore, cell viability of SAS was significantly reduced after 2 μM or 4 μM WA treatment (Fig 7B); this cell line was even more sensitive to WA than PC-3 or DU-145 (see Fig 1). c-Fos, HSPA6, and HSP70 were induced, while FLIP(L) was reduced, following WA treatment, and apoptotic markers (PARP and caspase-3) appeared 24 h after 2 μM or 4 μM WA treatment (Fig 7C). These results suggest that WA is herapeutically useful to cure the mCRPCs.

Like other CSC lines, SAS exhibits higher chemoresistance to cisplatin than adherent cells [37]. Hence, we asked whether SAS and PC-3 in SFM culture also exhibited higher chemoresistance to WA (Fig 7D-i and 7D-iii). Indeed, neither SAS nor PC-3 in SFM culture was more resistant to WA than adherent cells (Fig 7D-ii and 7D-iv), suggesting that WA efficiently overcomes the chemoresistance barrier of CSCs. This result is similar to that of a recent report that sphere formation protects against cell death induced by integrin inhibition [38]. Taken together, WA may be useful for the development of drugs that induce cell death in CSCs.

## Discussion

Here, we showed that 2 μM WA treatment induced cell death in PC-3 and DU-145, but not in TIG-1 or LNCaP (Fig 1); TIG-1 and KD remained resistant to 2 μM WA treatment for up to 96 h, but LNCaP died at 72 h (S1 Fig). We investigated transcriptome profiles to identify the molecular mechanisms that determine the differential sensitivity of PC-3/DU-145 and TIG-1/LNCaP to WA-induced cell death. We first noticed that WA increased both mRNA (S1 Table) and protein (Fig 2A) levels of c-Fos in PC-3 and DU-145, but not in TIG-1 or LNCaP (Fig 2A). As expected, suppression of c-Fos by siRNA (siFos) decreased necrotic cell death (Fig 2E-i). However, siFos increased the frequency of apoptotic cell death in PC-3 (Fig 2E-ii and 2E-iii), suggesting that WA-induced cell death is not solely due to c-Fos induction. Gene Ontology (S4 Fig) and western blot (Fig 4) analyses indicated that the ER stress response is activated in PC-3, suggesting that ER stress is responsible for cell death in PC-3, but not TIG-1 or LNCaP (Fig 4). WA also induced up-regulation of HSPs (S1 Table and Fig 3) and autophagy (Fig 5); these pathways appear to protect cells against WA-induced cell death. Moreover, LNCaP, PC-3, and DU-145, but not TIG-1 or KD, efficiently generated ROS after WA treatment (Fig 4B, S7 Fig), which may explain why TIG-1 and KD are resistant to WA.

TNF and TRAIL are proapoptotic factors in many cancers, but some tumors acquire resistance and decrease the clinical utility of these agents [38]. TRAIL-induced apoptosis is down-regulated by a mammalian cellular homologue of FLIP, whose transcription is repressed by direct binding of c-Fos to its promoter region [18]. A previous study showed that ectopic expression of c-Fos in LNCaP reduced FLIP, but did not promote cell death [18]; this conclusion is similar to ours, in that an increased c-Fos level and a reduced FLIP level are independent events caused by WA (Fig 2C and 2F). Thus, we propose that c-Fos is efficiently activated by WA in DU-145, but not in TIG-1 or LNCaP, which indirectly leads to a reduced level of FLIP.

We also observed differences in the reorganization of the vimentin and F-actin cytoskeletons following WA treatment in PC-3 (Fig 6B-i and 6B-ii) relative to TIG-1. Specifically, vimentin and F-actin formed colocalized aggregates in TIG-1 (Fig 6B-i), whereas these aggregates were absent in PC-3 (Fig 6B-ii). Disorganization of the F-actin cytoskeleton has also been reported in frog kidney cells [28]. The partial rescue of cell viability by the actin-stabilizing toxin, jasplakinolide, suggests that WA-induced dysregulation of F-actin is at least partially responsible for WA-induced apoptosis (Fig 6D-ii). Moreover, immuno-electron microscopy using anti-vimentin antibody revealed that cytoskeletal structure was disrupted in PC-3 following WA treatment (S5 Fig). Exposure of WI-38 human diploid lung fibroblasts to cytochalasin B, a membrane-permeable inhibitor of actin polymerization, also antagonizes WA-induced cytotoxicity [39], likely because WA binds covalently to the Annexin A2 core domain, stimulating its basal F-actin cross-linking activity and ultimately causing alterations in the cytoskeletal architecture [40], [41]. These reports support our conclusion and suggest that the insult to cytoskeletal architecture caused by WA may result in the induction of HSPs and c-Fos, thereby leading to ROS generation and apoptosis.

Many cancer patients experience recurrence following chemotherapy. CSCs within the tumor mass are proposed to mediate this chemoresistance, and this capability to regenerate tumors distinguishes CSCs from other cancer cells [42]. The mechanisms of CSC chemoresistance identified to date include elevated ATP-binding cassette (ABC) transporter activity, aldehyde dehydrogenase (ALDH) activity, and enhancement of the DNA damage response [43]. PC-3 and SAS, which possess CSC-like properties [21], [22] exhibit higher chemoresistance to cisplatin as spheroids than as adherent cells (Fig 7D-i and 7D-ii) [37]. By contrast, PC-3 and SAS exhibited similar chemoresistance to WA as spheroids and adherent cells (Fig 7D-ii and 7D-iv).

Taken together, these data demonstrate that WA targets vimentin and promotes cell death in prostate cancer cells, but not in normal fibroblasts, through increased c-Fos level, reduced FLIP level, and/or enhanced ROS generation probably via WA-mediated disruption of the cytoskeletal architecture. Thus, WA may serve as a starting molecule for the development of drugs that effectively kill CSCs.

## Materials and Methods

### Antibodies and reagents

Antibodies against the following proteins were purchased from the indicated companies: α-Tubulin (Sigma-Aldrich); BAG3 (OriGene); BiP, Caspase-3 (8G10), c-Fos, CHOP, EGR-3, eIF2α, eIF2αx-pS51, FLIP (D16A8), FosB, HSF-1, HSP40, HSP70, IRE1α, PARP, PAR-4, PERK, TAZ, and YAP (Cell Signaling); EGR-1 and Withaferin A (Santa Cruz Biotechnology); GAPDH (Fitzgerald); HSPA6 (Abcam); LC3 (MBL); and vimentin (ARP).

### Cell culture

Human cells were acquired from the indicated suppliers: PC-3 and DU-145 (RIKEN Bioresource Center), TIG-1 (Japanese Cancer Research Resources Bank), KD (Japanese Collection of Research Bioresources Cell Bank) and LNCaP (ATCC). TIG-1 was maintained in 5% CO_2_ at 35°C in DMEM supplemented with FBS (10%), penicillin (100 U/mL), and streptomycin (100 μg/mL). For PC-3, DU-145, and LNCaP, DMEM was replaced with RPMI-1640 medium.

### siRNA

The following synthesized siRNA duplexes were used for siRNA-mediated knockdown: siGL2, CGUACGCGGGAAUACUUCGADdTdT (Gene Design); siHSPA6, GGCAGAGAAGGAGGAGUAUGAGCdAdT (OriGene); siHSF-1, GAGUGAAGACAUAAAGAUCCGCCdAdG (OriGene); siFos, GCAUUAACUAAUCUAUUGGGUUCdAdT (OriGene); siBAG3, CCUGAUGAUCGAAGAGUAUUUGAdCdC (OriGene); and CHOP, AGCGUAUCAUGUUAAAGAUGAGCGG (OriGene). These siRNA duplexes were transfected using Oligofectamine (Invitrogen).

## Supporting Information

S1 Fig Viability of TIG-1, KD, and LNCaP after WA treatment. Cell viability was measured at 4, 24, 48, 72, and 96 h after 2 μM WA treatment. NT, non-treated. Bars represent means ± SEM of three independent measurements.
(TIF)

S2 Fig Scatter plots of highlighted genes in S1 Table. The y-axis shows the log value of hybridization signal intensity obtained from the microarray data for WA-treated TIG-1, LNCaP, PC-3, and DU-145 (see yellow arrows in Fig 1B). The x-axis shows the log value of signal intensity obtained from samples treated with dimethyl sulfoxide (solvent). Dots corresponding to c-Fos, FosB, vimentin (VIM), and Par-4 are indicated by red, turquoise, black, and green arrows, respectively.
(TIF)

S3 Fig FACS pattern of PC-3 cells treated with or without siFos and/or WA. FACS analysis of PC-3 cells transfected with siFos or siGL2 (negative control) after treatment with WA (4 μM) or DMSO (solvent) alone, using the GFP-Certified Apoptosis/Necrosis Detection System. Apoptosis was detected by Annexin V–EnzoGold, and necrosis by 7-AAD-Red. Upper left, necrosis; upper right, late apoptosis; bottom right, early apoptosis.
(TIF)

S4 Fig Protein levels of EGR-1, EGR-3, and PAR-4 are constant following WA treatment. Western blot analysis for EGR-1, EGR-3, PAR-4, and GAPDH in PC-3 cells either untreated (NT) or 4, 8, or 24 h after treatment with 4 μM WA.
(TIF)

S5 Fig Gene Ontology analysis. (A) Genes differentially expressed between PC-3 cells treated with DMSO or 2 μM WA (see Fig 1B) were subjected to NextBio analysis to identify biogroups and studies (biosets) that contain similar genes. List of top five biogroups: “Biogroup name” signifies a collection of genes associated with a specific biological function, pathway, or similar criteria. ER stress–related biogroups are highlighted in red font. (B, C) Venn diagrams and bar graphs of “Response to unfolded protein” (B) and “Response to topologically incorrect protein” (C). Venn diagrams show the number of common and unique genes in both biosets and biogroups. “Common genes” indicate the number of overlapping genes between the bioset and biogroup. Bars at right indicate the significance of the overlap between gene subsets. The scale of the bar is-log (*p*-value), i.e., the taller the bar, the higher the significance of the overlap.
(TIF)

S6 Fig CHOP plays an essential role in the activation of caspase 3. Western blot analysis of CHOP, caspase 3, and GAPDH (loading control) in TIG-1 and PC-3 at 8 h after 4 μM WA treatment in the presence (+) or absence (-) of the indicated siRNAs. The arrow and arrowhead indicate the CHOP and caspase 3 band, respectively.
(TIF)

S7 Fig Observation of ROS signals under a fluorescence microscope. (A) Typical fluorescence images of TIG-1 (i), KD (ii), LNCaP (iii), PC-3 (iv), and DU-145 (v) that were cultured on glass coverslips and treated with TBHP for 90 min to induce ROS generation. Merged images are shown of cytoplasmic ROS signals (green) and nuclear DNA signals stained with Hoechst33342 (blue). Black bar, 10 μm. (B) Typical fluorescence images of KD cells that were incubated with 4 μM WA for 24 h and then treated with ROS detection reagents using the Image-iT LIVE Green ROS Detection Kit. To highlight the weak ROS signals (i), the image of nuclear DNA signals stained with Hoechst33342 (ii) is separately shown. Green bar, 100 μm.
(TIF)

S8 Fig Anti-vimentin immuno-electron microscopy of TIG-1 and PC-3 before and after WA treatment. Typical images obtained by immuno-electron microscopy of TIG-1 (A, B) and PC-3 (C, D) cultured for 4 h in the absence (A, C) or presence (B, D) of 4 μM WA. Multiple magnifications are shown (i–iii). Scale bars all indicate 1 μm in the corresponding images.
(TIF)

S1 File Supplementary results, materials, and methods. (DOCX)

S1 Table List of up-regulated genes following treatment with 4 μM WA treatment. Genes were arranged in descending order of fold change in PC-3; only the genes with a fold change > 4 in DU-145 are listed. HSP-related, EGR-related, Fos-related, and BAG genes are highlighted in red, green, blue, and pink font, respectively.
(TIF)

## References

[1] Hahm ER, Moura MB, Kelley EE, Van Houten B, Shiva S, Singh SV (2011) Withaferin A-induced apoptosis in human breast cancer cells is mediated by reactive oxygen species. PLoS One 6: e23354 10.1371/journal.pone.0023354 21853114 PMC3154436

[2] Zhang X, Mukerji R, Samadi AK, Cohen MS (2011) Down-regulation of estrogen receptor-alpha and rearranged during transfection tyrosine kinase is associated with withaferin a-induced apoptosis in MCF-7 breast cancer cells. BMC Complement Altern Med 11: 84 10.1186/1472-6882-11-84 21978374 PMC3198756

[3] Nagalingam A, Kuppusamy P, Singh SV, Sharma D, Saxena NK (2014) Mechanistic elucidation of the antitumor properties of withaferin a in breast cancer. Cancer Res 74: 2617–2129. 10.1158/0008-5472.CAN-13-2081 24732433 PMC4009451

[4] Choi MJ, Park EJ, Min KJ, Park JW, Kwon TK (2011) Endoplasmic reticulum stress mediates withaferin A-induced apoptosis in human renal carcinoma cells. Toxicol In Vitro 25: 692–698. 10.1016/j.tiv.2011.01.010 21266191

[5] Yang ES, Choi MJ, Kim JH, Choi KS, Kwon TK (2011) Withaferin A enhances radiation-induced apoptosis in Caki cells through induction of reactive oxygen species, Bcl-2 downregulation and Akt inhibition. Chem Biol Interact 190: 9–15. 10.1016/j.cbi.2011.01.015 21256832

[6] Yu Y, Hamza A, Zhang T, Gu M, Zou P, Newman B, et al (2010) Withaferin A targets heat shock protein 90 in pancreatic cancer cells. Biochem Pharmacol 79: 542–551. 10.1016/j.bcp.2009.09.017 19769945 PMC2794909

[7] Munagala R, Kausar H, Munjal C, Gupta RC (2011) Withaferin A induces p53-dependent apoptosis by repression of HPV oncogenes and upregulation of tumor suppressor proteins in human cervical cancer cells. Carcinogenesis 32: 1697–1705. 10.1093/carcin/bgr192 21859835

[8] Roy RV, Suman S, Das TP, Luevano JE, Damodaran C (2013) Withaferin A, a steroidal lactone from Withania somnifera, induces mitotic catastrophe and growth arrest in prostate cancer cells. J Nat Prod 76: 1909–1915. 10.1021/np400441f 24079846 PMC4144448

[9] Mayola E, Gallerne C, Esposti DD, Martel C, Pervaiz S, Larue L, et al (2011) Withaferin A induces apoptosis in human melanoma cells through generation of reactive oxygen species and down-regulation of Bcl-2. Apoptosis 16: 1014–1027. 10.1007/s10495-011-0625-x 21710254

[10] Bargagna-Mohan P, Hamza A, Kim YE, Khuan Abby Ho Y, Mor-Vaknin N, et al (2007) The tumor inhibitor and antiangiogenic agent withaferin A targets the intermediate filament protein vimentin. Chem Biol 14: 623–634. 17584610 10.1016/j.chembiol.2007.04.010 PMC3228641

[11] Bargagna-Mohan P, Deokule SP, Thompson K, Wizeman J, Srinivasan C, Vooturi S, et al (2013) Withaferin A effectively targets soluble vimentin in the glaucoma filtration surgical model of fibrosis. PLoS One 8: e63881 10.1371/journal.pone.0063881 23667686 PMC3648549

[12] Grin B, Mahammad S, Wedig T, Cleland MM, Tsai L, Herrmann H, et al (2012) Withaferin a alters intermediate filament organization, cell shape and behavior. PLoS One 7: e39065 10.1371/journal.pone.0039065 22720028 PMC3376126

[13] ThaiparambilJT, BenderL, GaneshT, KlineE, PatelP, LiuY, et al (2011) Withaferin A inhibits breast cancer invasion and metastasis at sub-cytotoxic doses by inducing vimentin disassembly and serine 56 phosphorylation. Int J Cancer 129: 2744–2755. 10.1002/ijc.25938 21538350

[14] Lee J, Hahm ER, Marcus AI, Singh SV (2013) Withaferin A inhibits experimental epithelial-mesenchymal transition in MCF-10A cells and suppresses vimentin protein level in vivo in breast tumors. Mol Carcinog 2013 11 30. 10.1002/mc.22110 PMC4039625 24293234

[15] Antony ML, Lee J, Hahm ER, Kim SH, Marcus AI, Kumari V, et al (2014) Growth Arrest by the Antitumor Steroidal Lactone Withaferin A in Human Breast Cancer Cells Is Associated with Down-regulation and Covalent Binding at Cysteine 303 of β-Tubulin. J Biol Chem 289: 1852–1865. 10.1074/jbc.M113.496844 24297176 PMC3894360

[16] Smeyne RJ, Vendrell M, Hayward M, Baker SJ, Miao GG, Schilling K, et al (1993) Continuous c-fos expression precedes programmed cell death in vivo. Nature 363: 166–169. 8483500 10.1038/363166a0

[17] Preston GA, Lyon TT, Yin Y, Lang JE, Solomon G, Annab L, et al (1996) Induction of apoptosis by c-Fos protein. Mol Cell Biol 16: 211–218. 8524298 10.1128/mcb.16.1.211 PMC230994

[18] Zhang X, Zhang L, Yang H, Huang X, Otu H, Libermann TA, et al (2007) c-Fos as a proapoptotic agent in TRAIL-induced apoptosis in prostate cancer cells. Cancer Res 67: 9425–9434. 17909052 10.1158/0008-5472.CAN-07-1310 PMC2941899

[19] Cereda V, Formica V, Massimiani G, Tosetto L, Roselli M (2014) Targeting metastatic castration-resistant prostate cancer: mechanisms of progression and novel early therapeutic approaches. Expert Opin Investig Drugs 223: 469–487. 10.1517/13543784.2014.885950 24490883

[20] Fan X, Liu S, Su F, Pan Q, Lin T (2012) Effective enrichment of prostate cancer stem cells from spheres in a suspension culture system. Urol Oncol 30: 314–318. 10.1016/j.urolonc.2010.03.019 20843707

[21] Sheng X, Li Z, Wang DL, Li WB, Luo Z, Chen KH, et al (2013) Isolation and enrichment of PC-3 prostate cancer stem-like cells using MACS and serum-free medium. Oncol Lett 5: 787–792. 23426586 10.3892/ol.2012.1090 PMC3576206

[22] Wang L, Huang X, Zheng X, Wang X, Li S, Zhang L, et al (2013) Enrichment of prostate cancer stem-like cells from human prostate cancer cell lines by culture in serum-free medium and chemoradiotherapy. Int J Biol Sci 9: 472–479. 10.7150/ijbs.5855 23781140 PMC3677682

[23] Geary LA, Nash KA, Adisetiyo H, Liang M, Liao CP, Jeong JH, et al (2014) CAF-secreted Annexin A1 Induces Prostate Cancer Cells to Gain Stem Cell-like Features. Mol Cancer Res 12: 607–621. 10.1158/1541-7786.MCR-13-0469 24464914 PMC3989391

[24] Zhang K, Fowler M, Glass J, Yin H (2014) Activated 5'flanking region of NANOGP8 in a self-renewal environment is associated with increased sphere formation and tumor growth of prostate cancer cells. Prostate 74: 381–394. 10.1002/pros.22759 24318967

[25] Luo Y, Cui X, Zhao J, Han Y, Li M, Lin Y, et al (2014) Cells susceptible to epithelial-mesenchymal transition are enriched in stem-like side population cells from prostate cancer. Oncol Rep 31: 874–884. 10.3892/or.2013.2905 24316717

[26] Ciocca DR, Arrigo AP, Calderwood SK (2011) Heat shock proteins and heat shock factor 1 in carcinogenesis and tumor development: an update. Arch Toxicol 87: 19–48. 10.1007/s00204-012-0918-z PMC3905791 22885793

[27] Srinivasan S, Ranga RS, Burikhanov R, Han SS, Chendil D (2007) Par-4-dependent apoptosis by the dietary compound withaferin A in prostate cancer cells. Cancer Res 67: 246–253. 17185378 10.1158/0008-5472.CAN-06-2430

[28] Khan S, Rammeloo AW, Heikkila JJ (2012) Withaferin A induces proteasome inhibition, endoplasmic reticulum stress, the heat shock response and acquisition of thermotolerance. PLoS One 7: e50547 10.1371/journal.pone.0050547 23226310 PMC3511540

[29] Widodo N, Priyandoko D, Shah N, Wadhwa R, Kaul SC (2010) Selective killing of cancer cells by Ashwagandha leaf extract and its component Withanone involves ROS signaling. PLoS One 5: e13536 10.1371/journal.pone.0013536 20975835 PMC2958829

[30] Hahm ER, Singh SV (2013) Autophagy fails to alter withaferin a-mediated lethality in human breast cancer cells. Curr Cancer Drug Targets 13: 640–650. 23607597 10.2174/15680096113139990039 PMC3723758

[31] Gamerdinger M, Kaya AM, Wolfrum U, Clement AM, Behl C (2011) BAG3 mediates chaperone-based aggresome-targeting and selective autophagy of misfolded proteins. EMBO Rep 12: 149–156. 10.1038/embor.2010.203 21252941 PMC3049430

[32] Kabeya Y, Mizushima N, Ueno T, Yamamoto A, Kirisako T, Noda T, et al (2000) LC3, a mammalian homologue of yeast Apg8p, is localized in autophagosome membranes after processing. EMBO J 19: 5720–5728. 11060023 10.1093/emboj/19.21.5720 PMC305793

[33] Liu BQ, Du ZX, Zong ZH, Li C, Li N, Zhang Q, et al (2013) BAG3-dependent noncanonical autophagy induced by proteasome inhibition in HepG2 cells. Autophagy 9: 905–916. 10.4161/auto.24292 23575457 PMC3672299

[34] Ouyang DY, Xu LH, He XH, Zhang YT, Zeng LH (2013) Autophagy is differentially induced in prostate cancer LNCaP, DU145 and PC-3 cells via distinct splicing profiles of ATG5. Autophagy 9: 20–32. 10.4161/auto.22397 23075929 PMC3542215

[35] Shi Y, Tang B, Yu PW, Tang B, Hao YX, Lei X, et al (2012) Autophagy protects against oxaliplatin-induced cell death via ER stress and ROS in Caco-2 cells. PLoS One 7: e51076 10.1371/journal.pone.0051076 23226467 PMC3511352

[36] Lázaro-Diéguez F, Aguado C, Mato E, Sánchez-Ruíz Y, Esteban I, Alberch J, et al (2008) Dynamics of an F-actin aggresome generated by the actin-stabilizing toxin jasplakinolide. J Cell Sci 121: 1415–1425. 10.1242/jcs.017665 18398002

[37] Chen SF, Chang YC, Nieh S, Liu CL, Yang CY, Lin YS (2012) Nonadhesive culture system as a model of rapid sphere formation with cancer stem cell properties. PLoS One 7: e31864 10.1371/journal.pone.0031864 22359637 PMC3281010

[38] Silginer M, Weller M, Ziegler U, Roth P (2014) Integrin inhibition promotes atypical anoikis in glioma cells. Cell Death Dis 5:e1012 10.1038/cddis.2013.543 24457956 PMC4040659

[39] French LE, Tschopp J (1999) The TRAIL to selective tumor death. Nat Med 5: 146–147. 9930856 10.1038/5505

[40] Falsey RR, Marron MT, Gunaherath GM, Shirahatti N, Mahadevan D, Gunatilaka AA, et al (2006) Actin microfilament aggregation induced by withaferin A is mediated by annexin II. Nat Chem Biol 2: 33–38. 16408090 10.1038/nchembio755

[41] Ozorowski G, Ryan CM, Whitelegge JP, Luecke H (2012) Withaferin A binds covalently to the N-terminal domain of annexin A2. Biol Chem 393:1151–1163. 10.1515/hsz-2012-0184 23091278

[42] Kreso A, Dick JE (2014) Evolution of the Cancer Stem Cell Model. Cell Stem Cell 14: 275–291. 10.1016/j.stem.2014.02.006 24607403

[43] Abdullah LN, Chow EK (2013) Mechanisms of chemoresistance in cancer stem cells. Clin Transl Med 2: 3 10.1186/2001-1326-2-3 23369605 PMC3565873

