# Supplementary figures and images for: Withaferin A Induces Cell Death Selectively in Androgen-Independent Prostate Cancer Cells but Not in Normal Fibroblast Cells
Source: PLoS One. 2015 Jul 31;10(7):e0134137. doi: 10.1371/journal.pone.0134137 (PMC4521694; doi:10.1371/journal.pone.0134137)

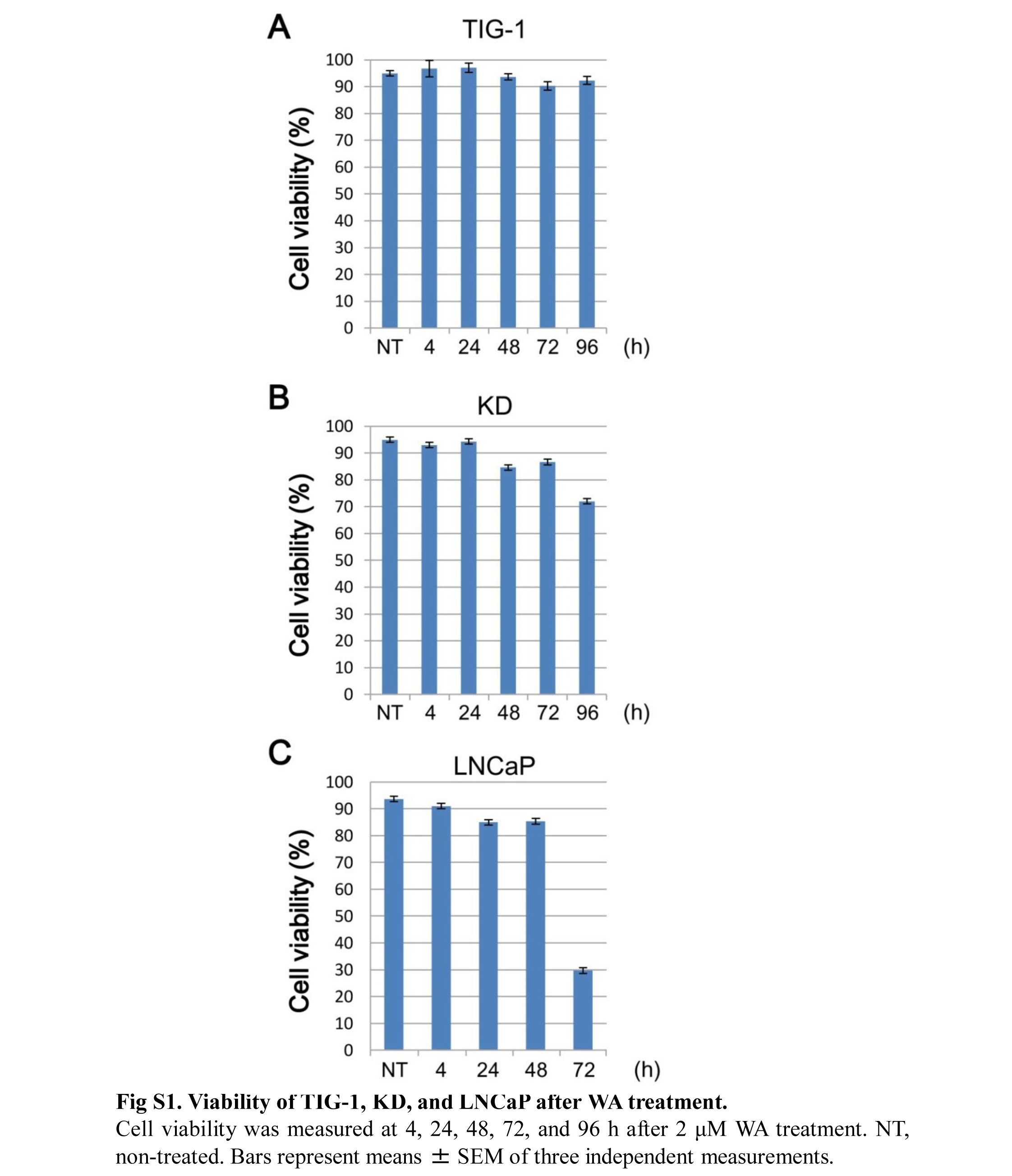

Supplement: S1 Fig — Cell viability was measured at 4, 24, 48, 72, and 96 h after 2 μM WA treatment. NT, non-treated. Bars represent means ± SEM of three independent measurements. (TIF) [file pone.0134137.s001.tif]

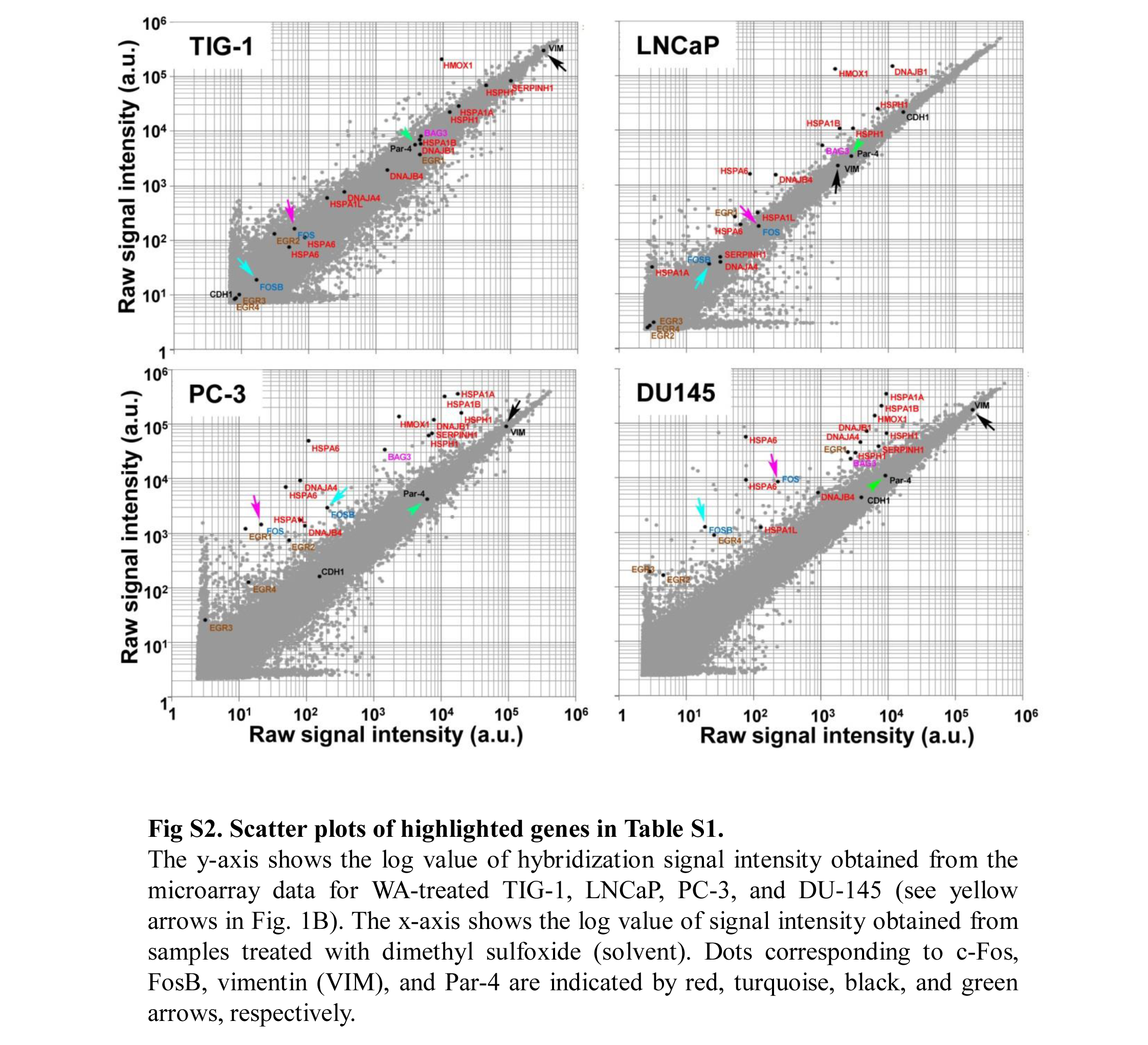

Supplement: S2 Fig — The y-axis shows the log value of hybridization signal intensity obtained from the microarray data for WA-treated TIG-1, LNCaP, PC-3, and DU-145 (see yellow arrows in Fig 1B). The x-axis shows the log value of signal intensity obtained from samples treated with dimethyl sulfoxide (solvent). Dots corresponding to c-Fos, FosB, vimentin (VIM), and Par-4 are indicated by red, turquoise, black, and green arrows, respectively. (TIF) [file pone.0134137.s002.tif]

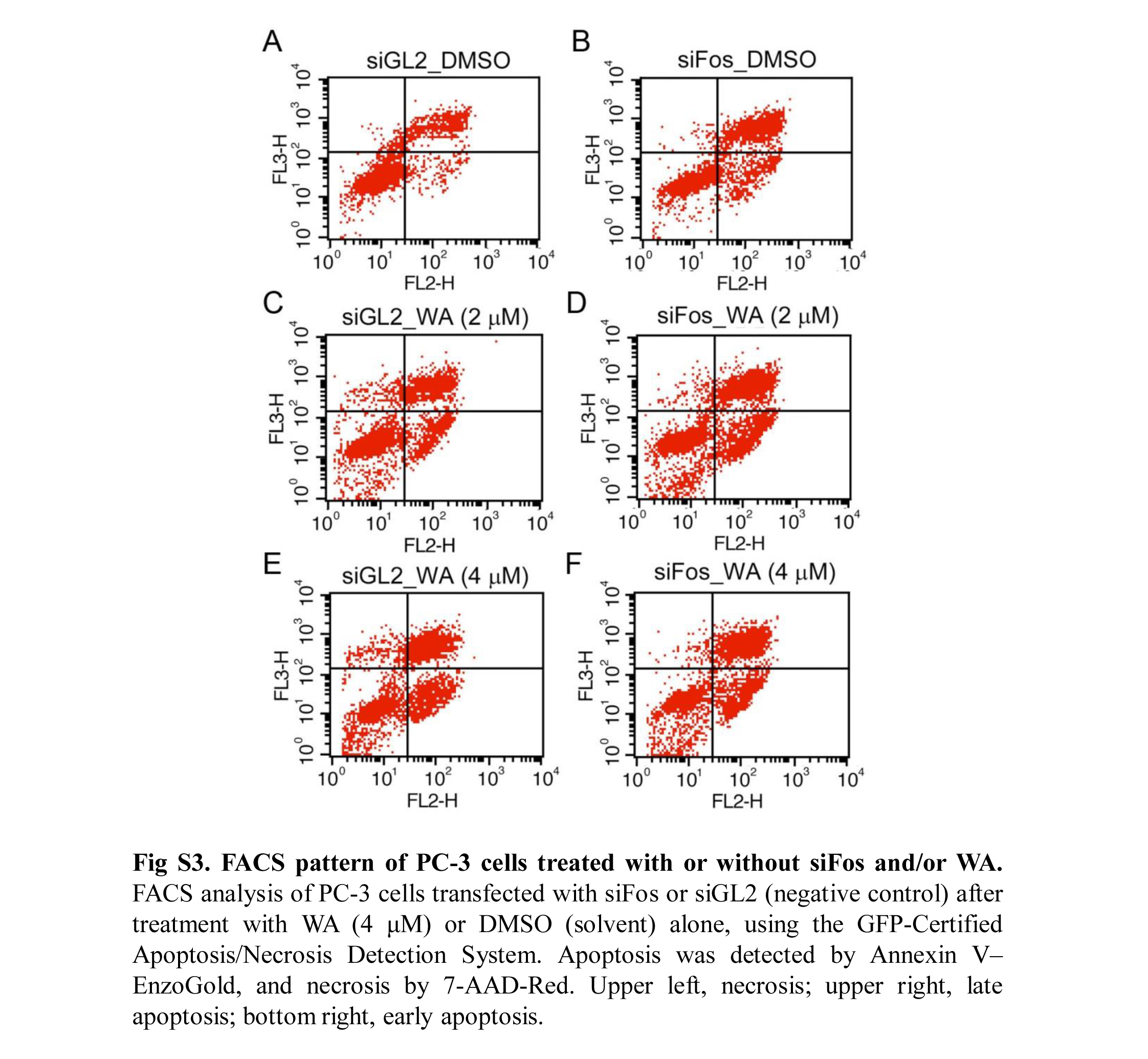

Supplement: S3 Fig — FACS analysis of PC-3 cells transfected with siFos or siGL2 (negative control) after treatment with WA (4 μM) or DMSO (solvent) alone, using the GFP-Certified Apoptosis/Necrosis Detection System. Apoptosis was detected by Annexin V–EnzoGold, and necrosis by 7-AAD-Red. Upper left, necrosis; upper right, late apoptosis; bottom right, early apoptosis. (TIF) [file pone.0134137.s003.tif]

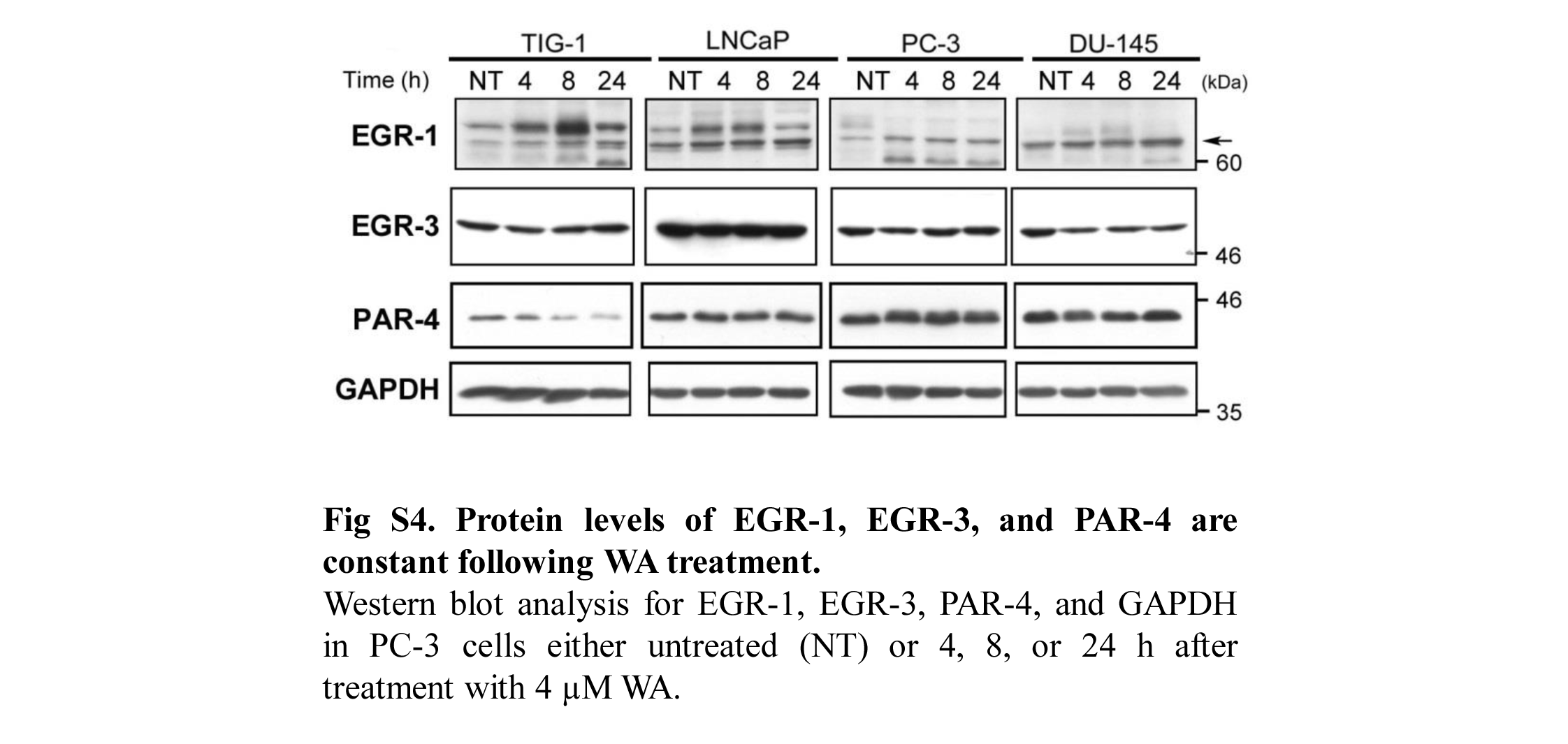

Supplement: S4 Fig — Western blot analysis for EGR-1, EGR-3, PAR-4, and GAPDH in PC-3 cells either untreated (NT) or 4, 8, or 24 h after treatment with 4 μM WA. (TIF) [file pone.0134137.s004.tif]

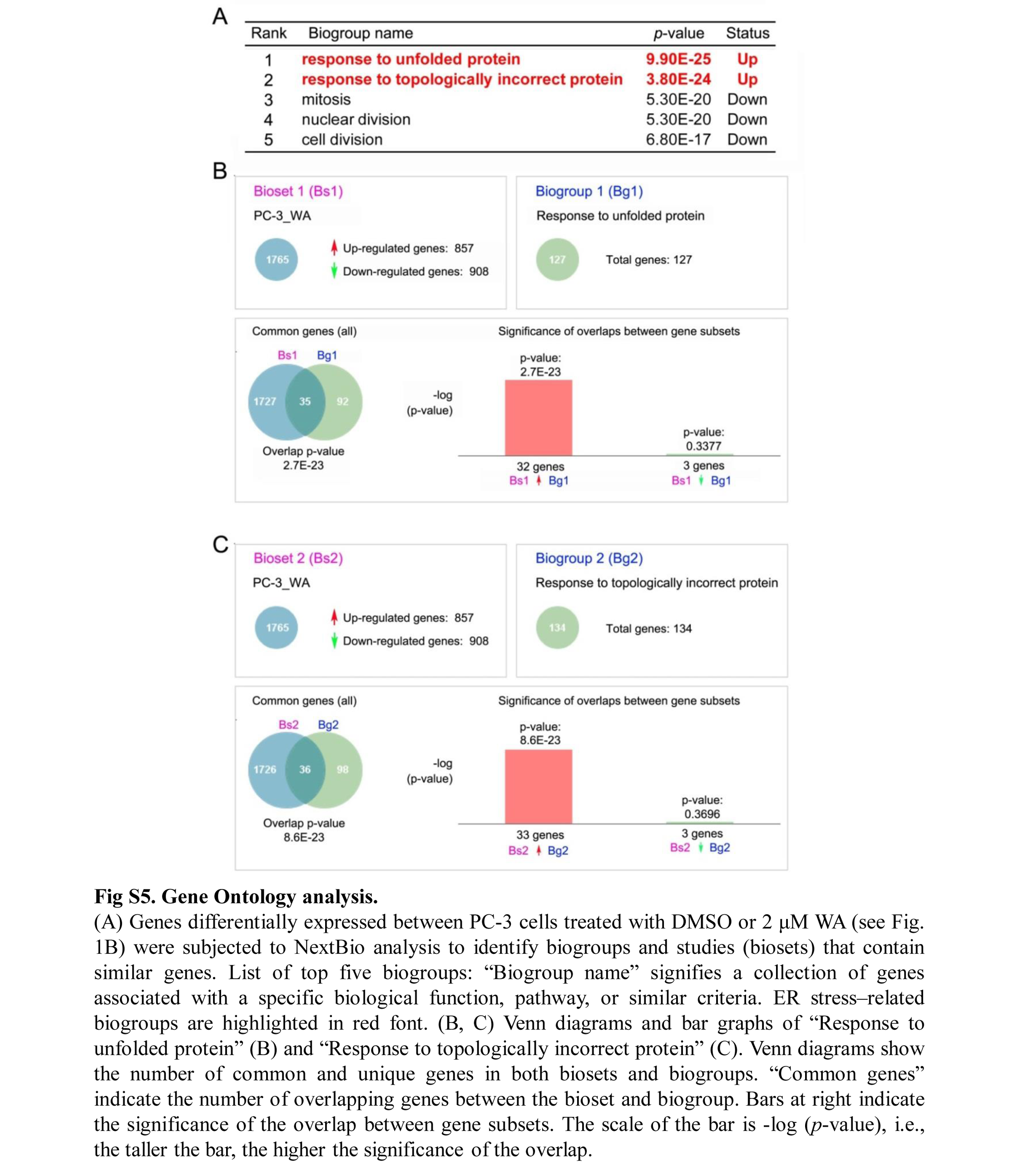

Supplement: S5 Fig — (A) Genes differentially expressed between PC-3 cells treated with DMSO or 2 μM WA (see Fig 1B) were subjected to NextBio analysis to identify biogroups and studies (biosets) that contain similar genes. List of top five biogroups: “Biogroup name” signifies a collection of genes associated with a specific biological function, pathway, or similar criteria. ER stress–related biogroups are highlighted in red font. (B, C) Venn diagrams and bar graphs of “Response to unfolded protein” (B) and “Response to topologically incorrect protein” (C). Venn diagrams show the number of common and unique genes in both biosets and biogroups. “Common genes” indicate the number of overlapping genes between the bioset and biogroup. Bars at right indicate the significance of the overlap between gene subsets. The scale of the bar is-log (p-value), i.e., the taller the bar, the higher the significance of the overlap. (TIF) [file pone.0134137.s005.tif]

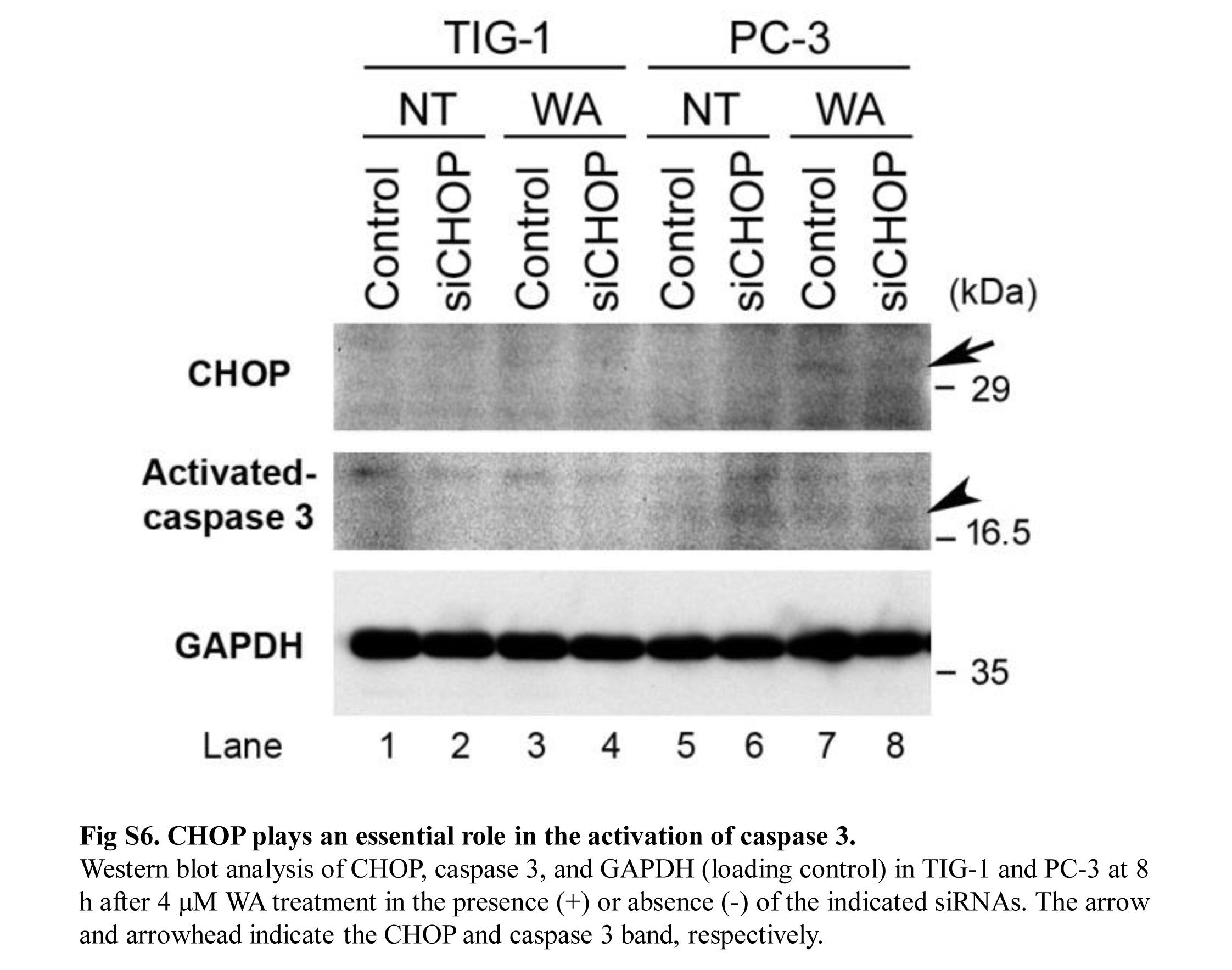

Supplement: S6 Fig — Western blot analysis of CHOP, caspase 3, and GAPDH (loading control) in TIG-1 and PC-3 at 8 h after 4 μM WA treatment in the presence (+) or absence (-) of the indicated siRNAs. The arrow and arrowhead indicate the CHOP and caspase 3 band, respectively. (TIF) [file pone.0134137.s006.tif]

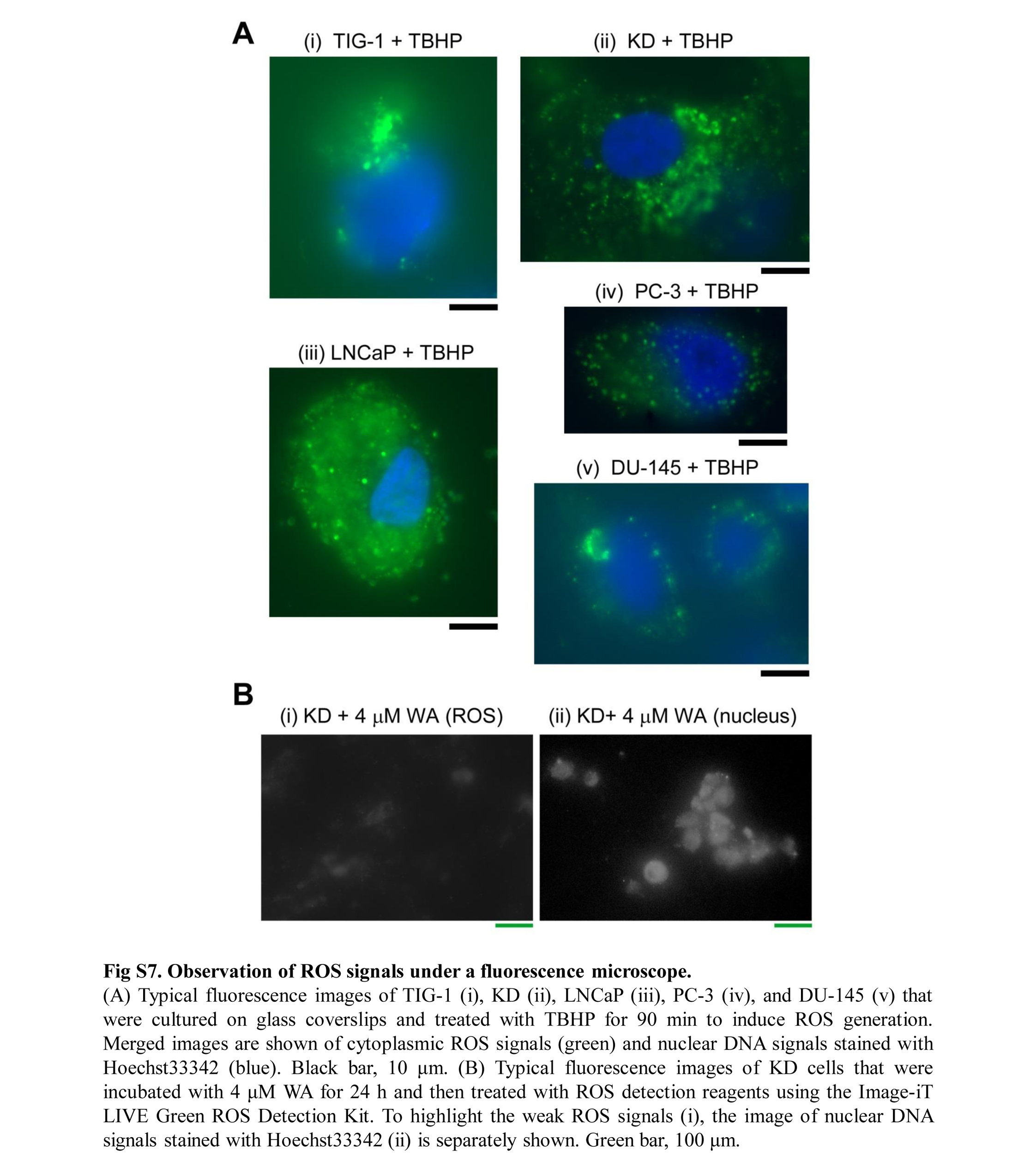

Supplement: S7 Fig — (A) Typical fluorescence images of TIG-1 (i), KD (ii), LNCaP (iii), PC-3 (iv), and DU-145 (v) that were cultured on glass coverslips and treated with TBHP for 90 min to induce ROS generation. Merged images are shown of cytoplasmic ROS signals (green) and nuclear DNA signals stained with Hoechst33342 (blue). Black bar, 10 μm. (B) Typical fluorescence images of KD cells that were incubated with 4 μM WA for 24 h and then treated with ROS detection reagents using the Image-iT LIVE Green ROS Detection Kit. To highlight the weak ROS signals (i), the image of nuclear DNA signals stained with Hoechst33342 (ii) is separately shown. Green bar, 100 μm. (TIF) [file pone.0134137.s007.tif]

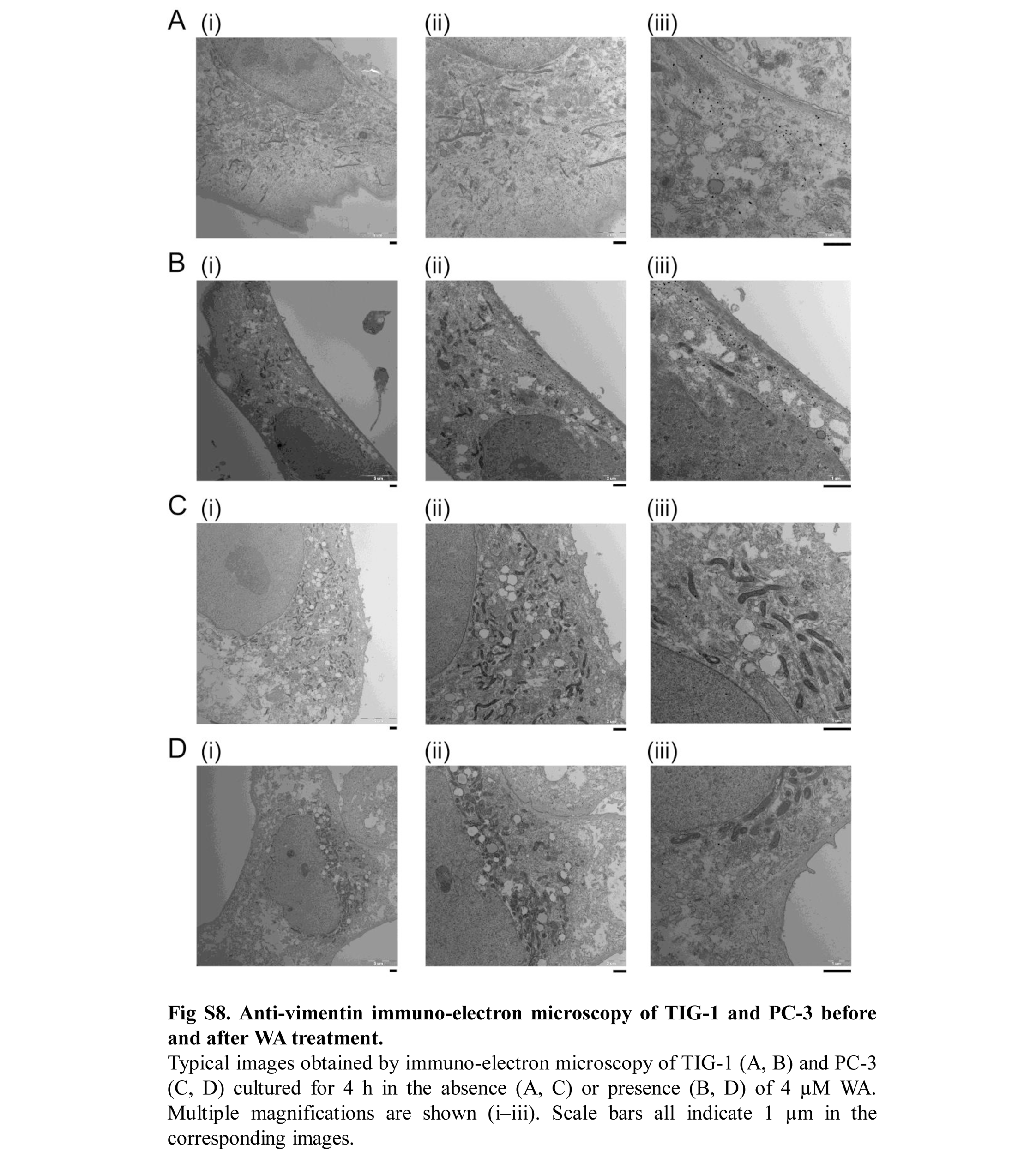

Supplement: S8 Fig — Typical images obtained by immuno-electron microscopy of TIG-1 (A, B) and PC-3 (C, D) cultured for 4 h in the absence (A, C) or presence (B, D) of 4 μM WA. Multiple magnifications are shown (i–iii). Scale bars all indicate 1 μm in the corresponding images. (TIF) [file pone.0134137.s008.tif]

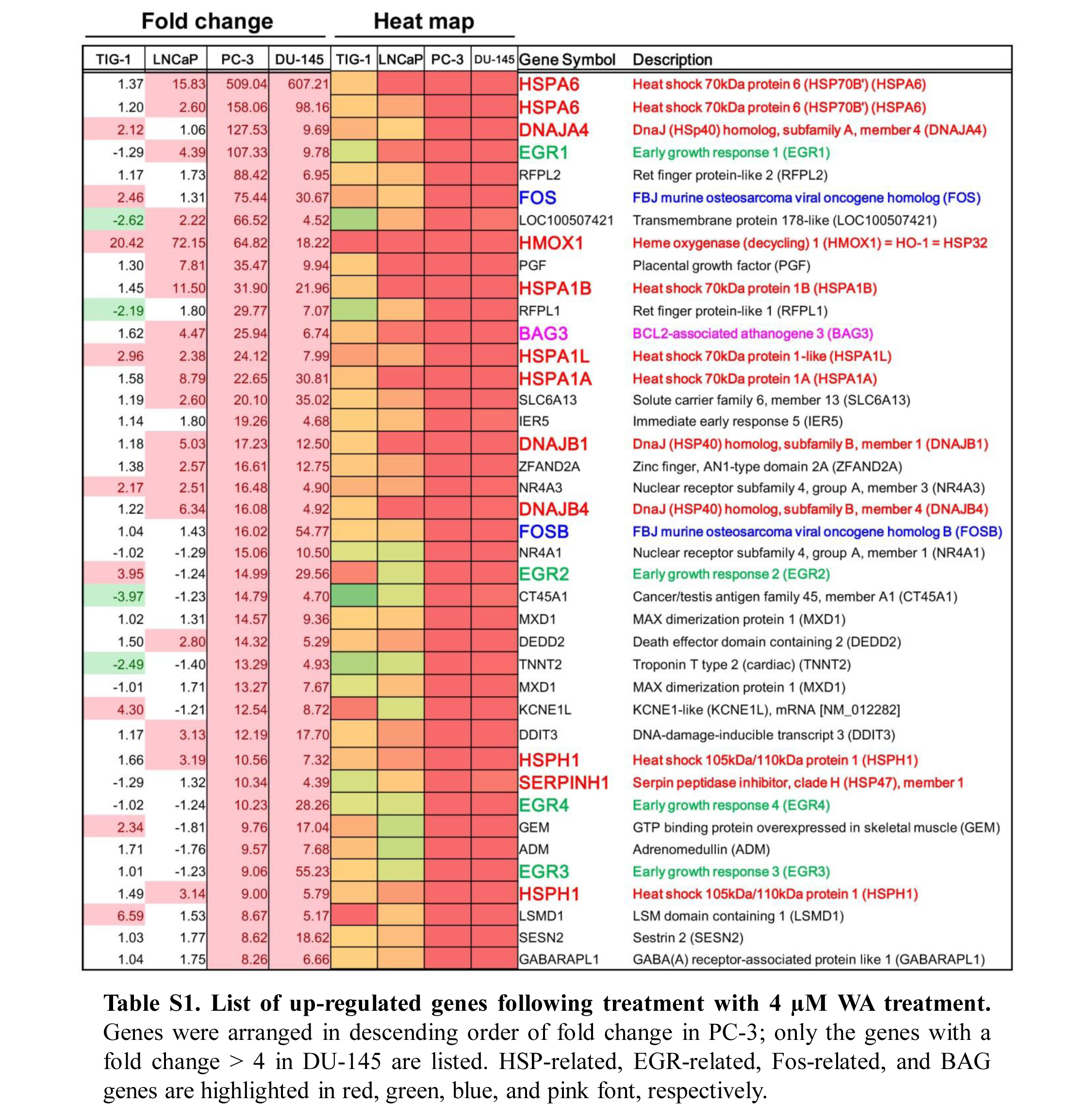

Supplement: S1 Table — Genes were arranged in descending order of fold change in PC-3; only the genes with a fold change > 4 in DU-145 are listed. HSP-related, EGR-related, Fos-related, and BAG genes are highlighted in red, green, blue, and pink font, respectively. (TIF) [file pone.0134137.s010.tif]
