# Supplementary material for: Withaferin A Induces Cell Death Selectively in Androgen-Independent Prostate Cancer Cells but Not in Normal Fibroblast Cells
Source: PLoS One. 2015 Jul 31;10(7):e0134137. doi: 10.1371/journal.pone.0134137 (PMC4521694; doi:10.1371/journal.pone.0134137)
Supplement: S1 File — (DOCX) [file pone.0134137.s009.docx]

**Supplementary Results**

**DNA microarray analysis**

To perform DNA microarray analysis, we selected the conditions for collection of samples at the timing when all or half of the cells are still alive. We selected two each sample at 2 μM and 4 μM. Because we wanted to identify the genes that allows TIG-1 and LNCaP cells resistant to WA treatment, we selected TIG-1 and LNCaP samples when they started to die. As for PC-3 and DU-145, we selected samples when the cell viability was about 50%.

**CHOP plays an essential role in the activation of caspase 3**

To investigate if there is any roles of enhanced CHOP expression in the activation of caspase 3, we performed siRNA-mediated knockdown of CHOP using siCHOP in TIG-1 (negative control) and PC-3. Western blot analysis confirmed the successful knockdown of CHOP by comparison of the band intensities between lanes 7 and 8 in Figure S6 (arrow). We found that the intensity of activated caspase 3 was almost the same between siControl and siCHOP (lanes 7 and 8). Moreover, the CHOP level was slightly induced by treatment with WA, but no increase of activated caspase 3 was observed (lanes 5 and 7). On the other hand, the amount of activated caspase 3 was increased by siCHOP without WA treatment (lanes 5 and 6), and this increase was more than that induced by co-treatment with siCHOP and WA (lanes 7 and 8). These results suggest that CHOP does not play a role in the activation of caspase 3.

**Supplementary Materials and Methods**

**Plasmid and reagents.**

Plasmid and reagents were purchased from the indicated companies: pEGFP-LC3 (Plasmid 21073) (Addgene); cisplatin (LKT Labs); 3-methyladenine, Earle’s Balanced Salts (EBSS), phalloidin, bFGF, EGF, Dulbecco’s modified Eagle’s medium (DMEM), medium, methyl tetrazolium (MTT) solution, and RPMI-1640 (Sigma–Aldrich); wortmannin, penicillin, leukemia inhibitory factor (LIF) (Millipore); and streptomycin and fetal bovine serum (FBS) (Hyclone).

**Cell viability.**

Cell viability was calculated as the number of viable cells divided by the total number of cells, which was measured by the trypan blue exclusion test using the Countess^TM^ Automated Cell Counter (Life Technologies).

**Spheroid formation assay.**

Single cells were plated in EZ-Bind ShutⅡ(AGC Techno Glass) at a density of 400 cells/cm^2^ and grown in sphere culture medium; for TIG-1 and SAS, serum-free DMEM/F-12 (1:1) was supplemented with B27 (Invitrogen), 20 ng/mL EGF, 20 ng/mL bFGF, penicillin (100 U/mL), and streptomycin (100 μg/mL); for PC-3, DU-145, and LNCaP, 20 ng/mL LIF was also added.

For spheroid formation assays, cells were mechanically dissociated using a 25 G injection needle (TERUMO) until doublets, triplets, and groups of cells were no longer observed. Single cells were plated in EZ-Bind Shut dishes at a density of 400 cells/cm^2^ and grown in sphere culture medium. Cells were analyzed microscopically after 1–5 d.

**Methyl tetrazolium (MTT) assay.**

To measure the *in vitro* cytotoxic effects of drugs by the MTT assay, PC-3 or SAS were plated at 1.0 × 10^4^ cells/well in 96-well adherent or non-adherent plates (EZ-Bind ShutⅡ) and incubated overnight. Then, 20 μL of WA or cisplatin was added to the indicated final concentration (see Fig. 7), and the cells were incubated for an additional 48 h. MTT assays were performed according to the manufacturer’s protocol (Life Technologies). Briefly, 10 μL MTT solution (5 mg/mL in PBS) was added, and cells were incubated for an additional 4 h. For cells in adherent plates, culture fluid was gently removed, 100 μL MTT solvent was added, and the samples were incubated for 30 min to allow the color change (from yellow to purple) to develop. For cells in non-adherent plates, cells were collected into 1.5 mL plastic tubes and centrifuged at 7,700 × *g* for 5 min; 100 μL MTT solvent was added to the pellet, and the sample was incubated for 30 min. The intensity of the color was measured using a spectrophotometer.

**Apoptosis/necrosis assay.**

Apoptosis and necrosis were detected using the GFP-Certified™ Apoptosis/Necrosis detection kit for microscopy and flow cytometry (Enzo Life Sciences).

**FACS analysis, western blotting, and immunofluorescence staining.**

Cells were stained using the CycleTEST PLUS DNA Reagent Kit (BD Bioscience). Analysis was performed using a FACS Calibur (BD Bioscience) with the CellQuest software. Preparation of whole-cell lysates, western blotting, and indirect immunofluorescence staining were performed as described (Yabuta et al., 2011).

**Exogenous expression of c-FLIP(L).**

DU-145 cells (1.2 × 10^6^ cells in a Φ60 mm dish) were transfected with pcDNA3-FLIP(L) or vector alone. Twenty-four hours after transfection, cells were treated with RPMI1640 containing 4 μM WA or DMSO (a negative control), and then cultured for an additional 12 h. After the cells were detached by trypsinization, collected, and stained with Trypan blue, they were counted on a Countess Automated Cell Counter (Invitrogen). Viability was assessed by counting c-FLIP(L)–expressing cells treated with WA or DMSO, and normalized to the viability of vector-expressing cells treated with DMSO.

**ROS detection.**

Production of ROS was detected by a fluorescent staining method using the Image-iTTM LIVE Green ROS Detection Kit (Molecular Probes Inc., USA) according to the manufacturer’s protocol. Briefly, TIG-1, KD, LNCaP, PC-3, and DU-145 were cultured on glass coverslips placed in 6-well plates, treated with TBHP for 90 min or 4 µM WA for 24 h, and then washed with Hanks’ Balanced Salt Solution (HBSS) three times, before glass slides were mounted for observation by fluorescence microscopy (BX51-34-FL; Olympus, Tokyo). LNCaP easily detached from coverslips and were therefore stained for ROS in a microfuge tube, spun down for washing with HBSS three times, dissolved in 30 µL of HBSS, and directly spread onto glass slides for observation by fluorescence microscopy.

**Immuno-electron microscopy.**

TIG-1 (in DMEM) and PC-3 (in RPMI-1640) were cultured overnight at ~5.0 × 10^4^ cells/well in a 24-well Celltight C-1 Cell Desk LF (Sumitomo Bakelite). The medium was replaced with fresh medium containing 0 or 4 µM WA, and the samples were cultured for an additional 4 h. The medium was removed, and the cells were fixed for 1 h with 4% formaldehyde in 0.1 M sodium-phosphate buffer (pH 7.4) and washed three times for 5 min in the same buffer. Cells were then permeabilized and blocked for 30 min with 0.2% saponin, 10% BSA, 10% normal goat serum, and 0.1% cold water fish-skin gelatin in the same buffer. Cells were stained with primary antibody (anti-vimentin monoclonal antibody, VIM3B4/ARP, 1:50 dilution) overnight at 4°C, washed six times for 10 min in the same buffer containing 0.005% saponin, and then stained for 2 h at room temperature with anti-mouse IgG antibodies conjugated to 1.4 nm gold particles (Nanogold-Fab' fragment of goat anti–mouse IgG; Cat. #2002, Nanoprobes), washed five times for 10 min in the same buffer containing 0.005% saponin, and washed once for an additional 10 min without saponin. Cells were then fixed for 10 min in 1% glutaraldehyde. Subsequently, cells were washed three times for 5 min in PBS containing 50 mM glycine, followed by three 5-min washes in PBS containing 1% BSA and three 5-min washes in Milli-Q water. Next, cells were treated with Goldenhance-EM (Nanoprobes) to increase the size of gold particles and allow for visualization by electron microscopy, and then washed with distilled water. Cells were post-fixed for 1 h with 1% osmium tetroxide and 1.5% potassium ferrocyanide in 0.1 M sodium-phosphate buffer (pH 7.4), dehydrated in graded series of ethanol, and embedded in Epon812 (TAAB Co. Ltd., UK). Finally, 80-nm ultra-thin sections of cells were stained with saturated uranyl acetate and Reynolds lead citrate solution. Electron micrographs were obtained on a JEM-1011 transmission electron microscope (JEOL, Japan).

**Microarray analysis.**

Microarray analyses were performed as single-color hybridizations using Agilent SurePrint G3 Human GE v2 8x60K Microarrays (Catalog-No. G4851B), as described previously (Nagi-Miura et al., 2013). Total RNA was extracted from TIG-1, LNCaP, PC-3, and DU-145 cells at the indicated times after treatment with WA (see yellow arrows in Fig. 1) using the miRNeasy Mini kit (Qiagen). The quality of the RNA was determined using the RNA 6000 Nano LabChip Kit on a Bioanalyzer 2100 (Agilent Technologies). RNA was reverse-transcribed using AffinityScript reverse transcriptase (Agilent Technologies) and oligo-dT primers containing the T7 RNA polymerase promoter sequence. *In vitro* transcription was then performed using T7 RNA polymerase and the Low input Quick-Amp Labeling Kit (Agilent Technologies) to label cRNAs with Cy3-CTP (Amersham Pharmacia Biotech, Piscataway, NJ). Purified Cy3-labeled cRNAs (600 ng) were hybridized to the microarrays. Washing, scanning, and gene analyses were performed according to the manufacturer’s protocol (Agilent Technologies). The Subio Platform and Subio Basic Plug-in (v1.15; Subio Inc., Aichi, Japan) was then used to calculate between-sample fold changes. Details of the microarray data have been deposited in the Gene Expression Omnibus (GEO; www.ncbi.nlm.nih.gov/geo) database (accession number GSE55728).

For ontological analysis, 1,762 genes that exhibited significant fold changes were categorized into 755 different biogroups from Gene Ontology (*p*-values < 0.05) using the NextBio system (ID, GO:0006986; Fig. S4).

**Statistical analysis.**

Error bars for all data represent standard deviations (SDs) from the mean. *P*-values were calculated using Student’s *t*-tests.
